# Population-level genome sequencing reveals distinct *Mycobacterium tuberculosis* intrahost mutational trajectories in simian immunodeficiency virus-coinfected and antiretroviral treated non-human primates

**DOI:** 10.1128/msystems.00329-26

**Published:** 2026-07-27

**Authors:** Michael C. Chao, Michael R. Chase, Shoko Wakabayashi, Andrew J. Vickers, Byron Roman, Forrest Hopkins, Peter H. Culviner, Maximillian G. Marin, Pauline Maiello, Collin R. Diedrich, Zandrea Ambrose, Philana Ling Lin, Qingyun Liu, Sarah M. Fortune

**Affiliations:** 1 Department of Immunology and Infectious Diseases, Harvard T. H. Chan School of Public Health 1857, Boston, Massachusetts, USA; 2 Department of Data Science, Dana-Farber Cancer Institute 1855, Boston, Massachusetts, USA; 3 Department of Microbiology and Molecular Genetics, University of Pittsburgh School of Medicine 12317, Pittsburgh, Pennsylvania, USA; 4 Center for Vaccine Research, University of Pittsburgh School of Medicine 588296, Pittsburgh, Pennsylvania, USA; 5 Department of Pediatrics, Children's Hospital of Pittsburgh of the University of Pittsburgh Medical Center 12317, Pittsburgh, Pennsylvania, USA; 6 Department of Genetics, University of North Carolina 312061, Chapel Hill, North Carolina, USA; Hangzhou Institute for Advanced Study, Hangzhou, Zhejiang, China; Chinese Academy of Agricultural Sciences, Beijing, China

**Keywords:** pathogenesis, DNA sequencing, evolution, *Mycobacterium tuberculosis*, tuberculosis, non-human primate, simian immunodeficiency virus, antiretroviral agents, dissemination

## Abstract

Whole-genome sequencing of *Mycobacterium tuberculosis* (Mtb) populations from clinical samples has increasingly identified genes undergoing selection within and between hosts that drive differential infection and treatment outcomes. However, the intrahost Mtb mutational landscape—especially in the context of human immunodeficiency virus (HIV) coinfection and antiretroviral therapy (ART)—remains less clear, as do the potential impacts of such mutations on Mtb infection dynamics. Here, we performed whole-genome sequencing of Mtb populations isolated from 477 infected tissues across 20 non-human primates (NHP), including animals co-infected with simian immunodeficiency virus (SIV) with or without virological suppression by ART. We identified 116 mutations that emerged during infection, including those that are overrepresented within individual tissues and a subset that are shared across tissues during Mtb dissemination. We further find differential mutation trajectories across treatment groups, with higher mutation frequency and bacterial outgrowth in SIV-infected hosts and increased prevalence of oxidative damage-associated mutations in coinfected animals on ART. Finally, we demonstrate a common pattern of mutation in Mtb lipid metabolism and polyketide synthase genes and identify a subset of NHP-derived mutations that have also independently arisen in human clinical isolates. Together, our population-based sequencing uncovers Mtb diversification during early infection, captures discrete bacterial dissemination events, and infers differential immune pressures faced by Mtb in the setting of SIV-Mtb coinfection and ART suppression.

**IMPORTANCE:**

Tuberculosis (TB) remains a leading cause of death worldwide, especially in people living with HIV (PLHIV). How HIV infection and antiretroviral therapy impact *Mycobacterium tuberculosis* (Mtb) intrahost evolution remains unclear. Using whole-genome sequencing from hundreds of infected tissues from non-human primates, we find that simian immunodeficiency virus coinfected hosts and those receiving antiretroviral therapy exert different immune pressures on Mtb, leading to differences in mutation rates and types of DNA damage that are incurred. Mtb mutations were enriched in genes involved in lipid metabolism, and some of these are also seen in human TB strains. This work highlights the role of immune pressure in altering bacterial pathways that may enable Mtb adaptation to the host.

## INTRODUCTION

Tuberculosis (TB) remains the top single infectious cause of mortality worldwide, with people living with HIV (PLHIV) bearing an outsized cost of the ongoing epidemic (1). Studies have shown significant improvement in preventing TB mortality in this population through the widespread use of antiretroviral therapy (ART)—especially when started early—but individuals on long-term ART still retain an elevated risk of developing active TB disease despite virologic control and stable CD4 counts (2–5). Though studies have identified dysfunctional adaptive and innate immune responses associated with HIV/Mtb coinfection (e.g., reviewed in reference 6), the mechanisms accounting for increased TB disease progression in PLHIV remain incompletely understood. To model early *Mycobacterium tuberculosis* (Mtb) infection outcomes related to HIV coinfection, a recent study in non-human primates (NHP) established chronic simian immunodeficiency virus (SIV) infection first—including a subset of animals with virologic suppression using ART—before challenging animals with a genetically barcoded isogenic library of Mtb (7). This work found that SIV-positive animals developed more severe TB disease, including higher bacterial burden and greater inflammation, and that early ART during SIV infection could significantly reduce these effects. However, PET-CT imaging, pathology scoring, and sequencing of barcoded Mtb strains also showed that ART did not restore Mtb dissemination to levels observed in non-SIV-infected controls—especially to extrapulmonary sites—suggesting that ART animals experienced continuing defects in certain aspects of early Mtb control (7). The impact of SIV and ART on the bacterial intrahost evolutionary landscape in this study also remained unclear.

Whole-genome sequencing of Mtb clinical strains is increasingly identifying genes and pathways undergoing selection in hosts that drive differential infection and treatment outcomes (8–10). These mutational signatures have been powerful tools for studying long-term co-evolution of Mtb with human hosts (10) and for the identification of novel mutations that contribute to the emergence of antibiotic resistance (11, 12), many of which have been translated into diagnostics for detection of multi- and extremely drug-resistant infections. Further, sequencing of intrahost Mtb populations can identify unfixed antibiotic resistance mutations that emerge during treatment (13–15) and can serve as a record of host immune pressure acting on Mtb (16, 17). However, sequencing is often limited to anatomically accessible sites such as the airway, with studies suggesting that there is greater Mtb diversity present in the human lung and extrapulmonary tissues compared to sputum (18, 19).

In this study, we hypothesized that sequencing Mtb genomes from anatomically diverse tissues in NHPs would comprehensively identify intrahost mutations, uncover distinct immune pressures in the context of SIV coinfection with and without ART, and also highlight biologic processes that may be important for Mtb adaptation to the host milieu. Here, we performed whole-genome sequencing of Mtb populations isolated from 477 infected tissues from 20 non-human primates, finding 116 Mtb mutations that arose within the host. These mutations show that the Mtb mutation frequency is significantly increased in SIV-infected NHPs, while the proportion of oxidative damage-associated mutations is increased in animals on ART compared to non-SIV controls. We further show that Mtb lipid metabolism and biosynthetic genes are enriched for intrahost mutations, which we propose reflect opportunities for Mtb adaptation to host nutrient conditions.

## RESULTS

### Whole-genome sequencing defines the intrahost Mtb mutational landscape

**Fig 1:**
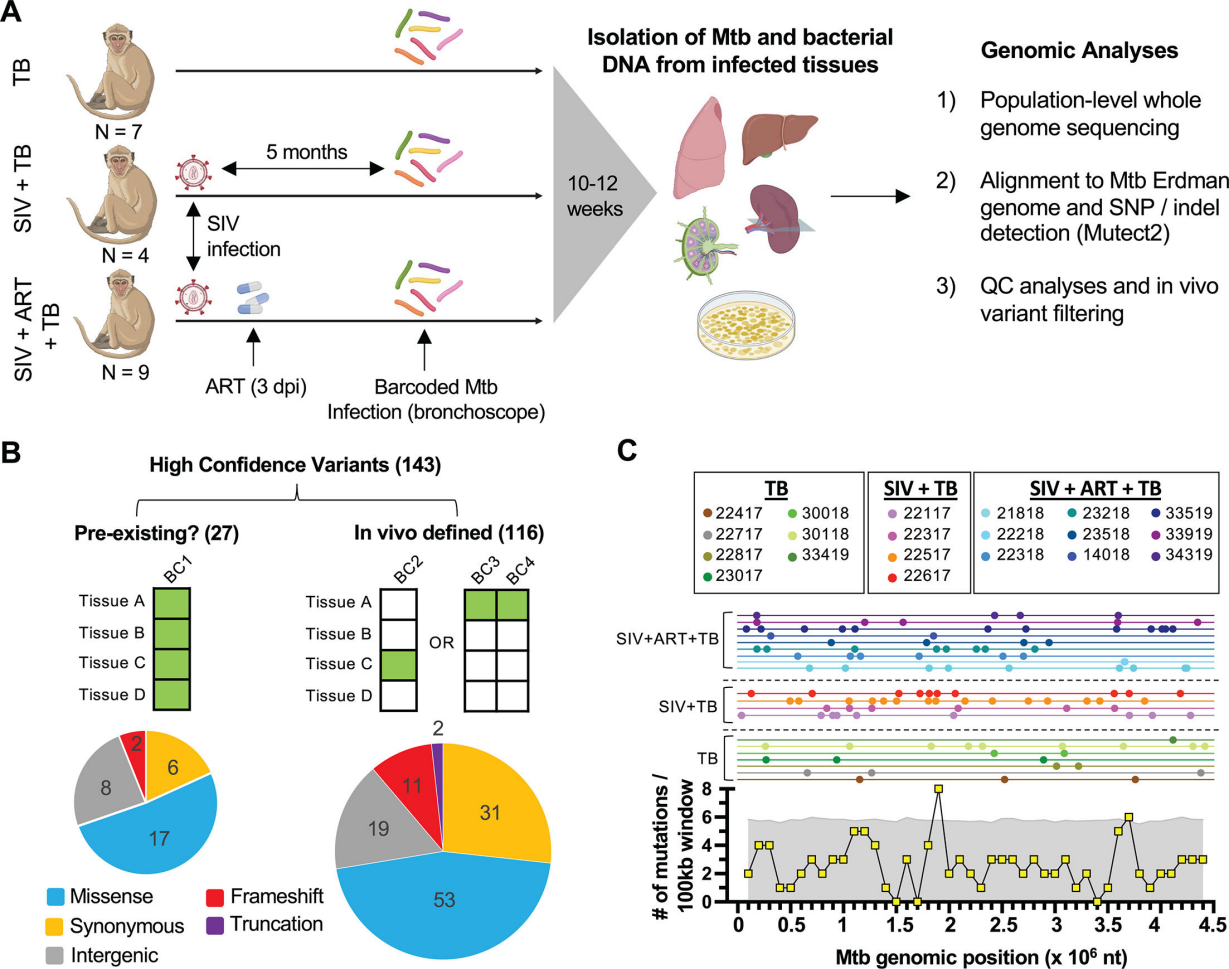
Whole-genome sequencing identifies intrahost mutations. (**A**) Study design in which naïve, SIV-infected NHPs (with and without suppressive ART initiated at 3 days post-infection) were challenged with a barcoded Mtb library. All bacteria were isolated from infected tissues, genomic DNA extracted for whole-genome sequencing, and intrahost mutations identified. (**B**) Top: criteria for defining *in vivo-*derived mutations, where every row (Tissues A–D) represents sites infected by a barcoded Mtb strain (columns BC1–4). Tissues containing a genomic mutation are colored green, while white indicates the presence of wild-type Mtb. Bottom: mutations are grouped by their predicted impact on protein coding. (**C**) Top: for each animal (lines), the genomic positions with detected mutations in any tissue are plotted (dots). Bottom: the numbers of mutations (from all NHPs) observed within 100-kb genomic windows are plotted (yellow). Gray shading indicates two standard deviations of the number of expected mutations in a given 100-kb window when sampling 116 random sites in the Mtb genome across 1,000 simulations. The simulation was conducted after masking the repetitive sites in the genome (as was done for intrahost mutation detection [20]) but did not otherwise consider other genomic features like nucleotide identity.

In this work, we curated a set of Mtb genomic DNA samples isolated from a prior non-human primate (NHP) study (7), which included naïve or chronically SIV-infected animals (with or without ART) that were challenged with a barcoded library of Mtb. In prior work, the viable Mtb populations from all infected tissues (including lung, thoracic lymph node, and extrapulmonary sites) were plated for genomic DNA extraction, and amplicon-based sequencing was used to define the barcoded Mtb strains that were infecting each tissue (7). In this study, we performed whole-genome sequencing of this DNA from a subset of 477 intrahost Mtb populations isolated from 20 NHPs (Table S1), including seven that were solely infected with Mtb (TB); four chronically infected with SIV prior to Mtb challenge (SIV + TB); and nine SIV infected but virally suppressed with ART (SIV + ART + TB) only 3 days after infection (Fig. 1A).

Mtb sequences were mapped to a recently generated Mtb Erdman reference genome (20), which was derived from a representative colony from the library used to infect these NHPs. Using the haplotype mutation calling tool Mutect2, we defined over 100,000 single-nucleotide polymorphisms (SNPs) and small insertions and deletions (indels) across all samples, with the vast majority at low frequency and likely representing sequencing artifacts (Fig. S1A). Subsequent filtering of low-coverage sites and considering only variants at >10% prevalence within tissues (see Materials and Methods) yielded 143 unique, high-confidence candidate mutations that were not present in our reference genome (Table S2). As some variants could have arisen during library construction *in vitro* (e.g., see “BC1” in Fig. 1B), we further compared the tissues that contained a genomic variant against previously generated barcoding data from the same samples to downselect 116 mutations that were likely *in vivo*-derived (see Materials and Methods) as they are only found in a subset of sites infected by the same barcoded Mtb strain (e.g., BC2, BC3, and BC4 in Fig. 1B). This left 27 mutations that we cannot rule out as “pre-existing” in the infecting library (Table S2), so we elected to exclude these from downstream analyses.

Of the intrahost mutations, 57% were predicted to change protein coding (53 missense mutations, 11 frameshift indels, and 2 in-frame internal deletions), 31% were predicted to be synonymous, and 19 mutations were located within intergenic regions (Fig. 1B). Cumulatively, intrahost mutations occurred across the Mtb genome, but certain genomic regions harbored more mutations than would be expected given completely random sampling of 116 sites (Fig. 1C). Specifically, we observed local mutational maxima between nucleotides 1.8–1.9 Mb and 3.6–3.7 Mb, with the highest mutational density found between 1.8 and 1.9 Mb, a region that includes several polyketide synthase genes (*pks7*, *pks8,* and *pks11*).

### SIV-infected and antiretroviral-treated NHPs harbor Mtb with divergent mutational trajectories

**Fig 2:**
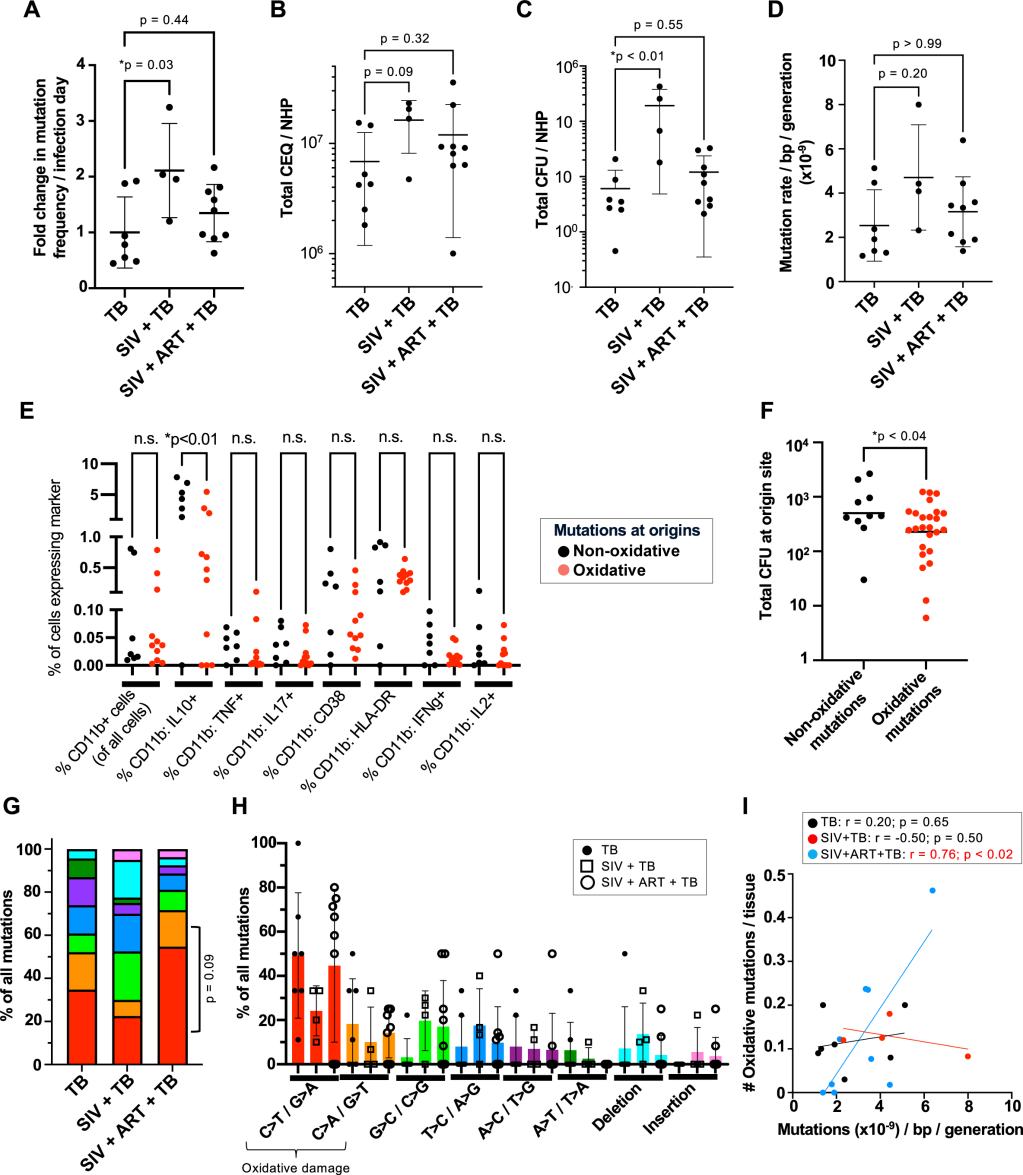
Mutational rates and profiles across SIV coinfection and ART. (**A**) Relative mutational frequencies were calculated for each animal per unit of infection time. (**B**) The total chromosomal equivalents (CEQs) from all infected tissues in each NHP were compared across treatment groups. (**C**) The total colony-forming units (CFUs) in each NHP at necropsy were compared across groups. (**D**) Mtb mutational rates per generation were calculated using an animal-specific generation time. (**A–D**) Statistical testing was performed using a Kruskal-Wallis test with Dunn’s correction. (**E**) Flow cytometry profiling of myeloid cells from origin lesions containing oxidative (C>T/G>A and C>A/G>T) and non-oxidative damage mutations (all other SNPs). Statistical comparisons were conducted using One-way ANOVA with Bonferroni correction. (**F**) The total Mtb CFUs at necropsy for origin sites from Mtb only and SIV + ART + TB NHPs containing an oxidative (C>T/G>A and C>A/G>T) or non-oxidative damage mutation (all other SNPs) were compared using an unpaired *t*-test. (**G**) The percent of all mutations observed in a treatment group was attributed to a distinct type of genomic mutation. *P* value was defined by Fisher’s exact test of oxidative mutations versus non-oxidative mutations in the ART group compared to the TB-only group. (**H**) The percent of all mutations in each NHP (dot) was separated by the type of mutation. (**I**) The correlation between the number of oxidative damage-associated mutations per NHP (normalized by the number of tissues sampled) with each animal’s calculated Mtb mutation rate per generation (one-tailed Pearson’s correlation).

We next calculated the relative Mtb mutation frequency across treatment groups using the total number of unique mutations identified in each animal (see Materials and Methods). First, we elected to use a fixed generation time for our calculations in order to define a relative mutation burden per infection day, which aimed to understand whether Mtb mutational frequencies were different across conditions. In Fig. 2A, we observe that SIV-coinfected NHPs (but not those on ART) had significantly higher mutational frequencies per day compared to Mtb-only animals. Next, we considered what may be possible drivers of a higher relative mutation frequency, which includes increased Mtb replication rates. In SIV-infected animals, there was a statistical trend (*P* = 0.09) of increased Mtb replication over the course of infection (Fig. 2B), as evaluated by quantifying chromosomal equivalents (CEQ)—the number of Mtb genomes in tissue homogenates representing the total live and dead bacteria burden over the course of infection. Further, there is significantly more live Mtb (CFU) in SIV-coinfected animals at necropsy (Fig. 2C), suggesting decreased capacity to clear Mtb.

To estimate the overall Mtb replication dynamics in the outbred NHP model and thereby evaluate the contribution of increased Mtb replication in driving Mtb diversification in SIV-coinfected NHPs, we first sought to determine the overall Mtb population doubling in each NHP by using the total unique barcodes (Fig. S1B) and CEQs (Fig. 2B) obtained across tissues in each animal as the starting and final Mtb population sizes, respectively (see Materials and Methods). These calculations found an average Mtb mutation rate of 2.5 × 10^−9^/bp/generation in the TB-only group (Fig. 2D), which is approximately 10-fold higher than the Mtb *in vitro* mutation rate that was previously calculated using fluctuation tests (16) and may reflect the contribution of host drivers of Mtb diversification. Next, we compared the intrahost replication dynamics across treatment conditions, finding that SIV-coinfected hosts support greater Mtb replication (Fig. S1C) and that Mtb have a significantly shorter generation time in SIV-coinfected hosts (Fig. S1D). When we now account for differences in the generation time, we do not observe a significant difference in the mutation rate per generation in SIV coinfected hosts (Fig. 2D), which suggests that the increased number of observed mutations in SIV-coinfected animals may be mainly driven by enhanced bacterial replication capacity in these immunocompromised hosts.

**Fig 3:**
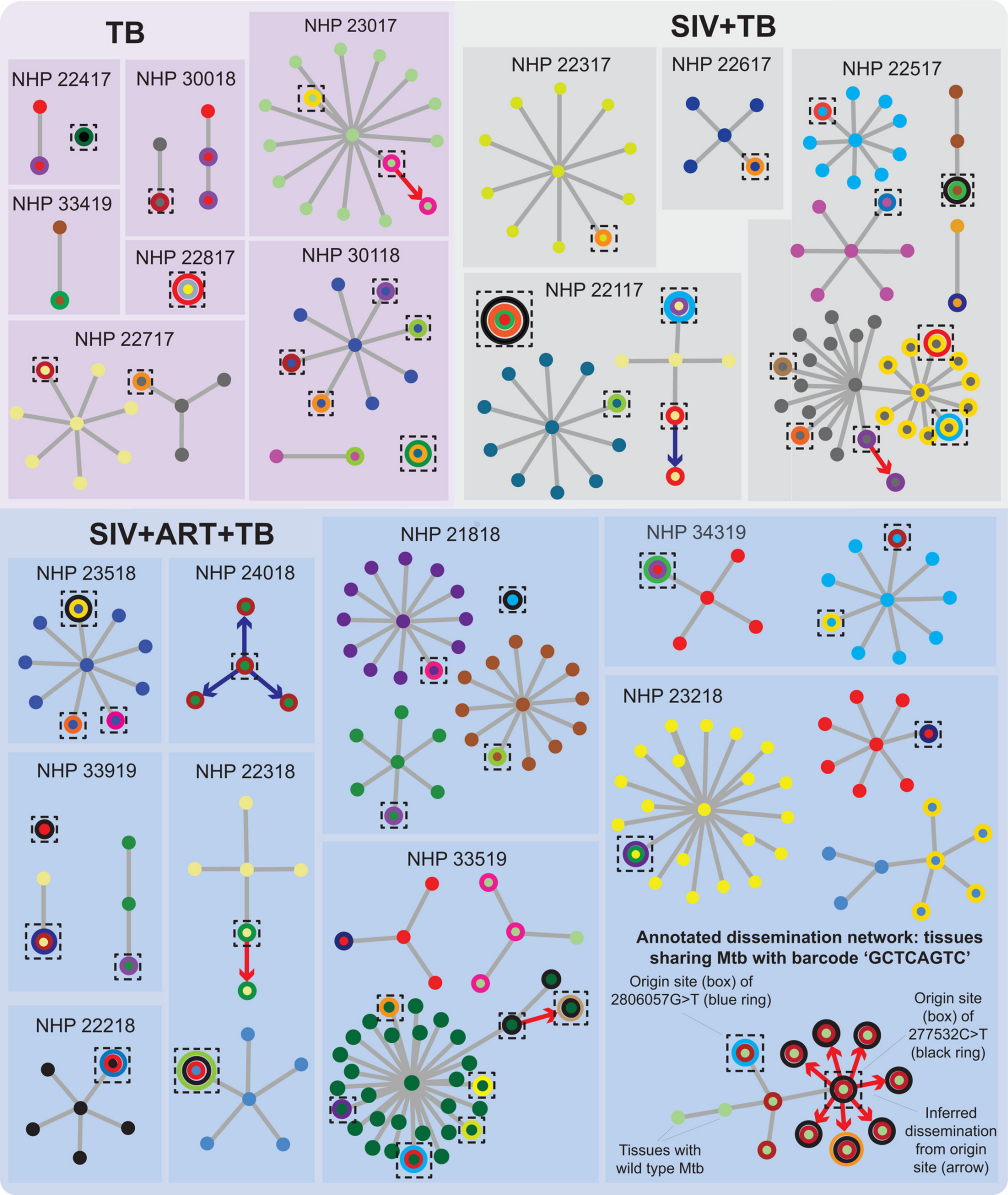
*In vivo* variants uncover discrete dissemination events within barcoded Mtb dissemination networks. Colored nodes denote tissues sharing the same Mtb strain, as defined from prior barcode sequencing in reference 7. Rings indicate Mtb mutations present in that tissue, with each color representing a different genomic mutation. Tissues with one barcoded Mtb strain but a mixture of wild-type and mutant alleles likely serve as “origin” sites, which are denoted as boxes. Inferred dissemination events from origin sites are indicated by arrows (red arrows indicate the origin site is a lung site disseminating to other lung tissues, while blue arrows indicate the origin site is a thoracic lymph node disseminating to lung tissues).

We next evaluated DNA damage due to host immune attack as another driver of intrahost mutation, as prior work suggested that the majority of intrahost Mtb mutations in latently infected NHPs and in sputum of active TB patients are mutations associated with oxidative damage (16, 17)—namely, C>T/G>A transitions and C>A/G>T transversions reflecting cytosine deamination (21) and 8-oxo-guanine formation (22), respectively. However, oxidative damage can also emerge *in vitro* during genomic DNA extraction (23), so we explored whether tissues harboring oxidative mutations are associated with markers of immune activation or the ability to control Mtb. To test these hypotheses, we first downselected 54 “origin” tissues (Fig. 3, boxed nodes) where genomic mutations likely arose (see Materials and Methods) as mutations found in some tissues could have hitchhiked through Mtb dissemination. Next, we found that 18 of these lesions (11 containing oxidative mutations and seven containing non-oxidative mutations) had been previously profiled by flow cytometry (7), so we assessed whether myeloid cells had differential expression of cytokines in oxidative and non-oxidative damage-associated lesions. In Fig. 2E, we observe that myeloid cells from oxidative mutation harboring lesions had significantly lower expression of IL-10, which is an immunomodulatory cytokine that has been shown to impair macrophage immune activation and Mtb control (24, 25). This may suggest that non-oxidative lesions have dampened capacity to control Mtb; and when we compared the Mtb burden at origin sites, we find that sites harboring oxidative mutations have significantly lower CFUs compared to sites associated with non-oxidative mutations (Fig. 2F). Thus, while it is difficult to causally link oxidative damage-associated mutations to immune attack without further interventional studies, our data are consistent with Mtb oxidative mutations arising in tissues associated with enhanced immune response.

We next compared the prevalence of oxidative mutations across treatment conditions, finding that SIV + ART + TB (but not SIV + TB) animals trended (*P* = 0.09) toward more oxidative mutations compared to Mtb-only animals (Fig. 2G). This signal was driven by a majority of ART animals harboring elevated frequencies of C>T/G>A and C>A/G>T mutations (Fig. 2H). Further, there is a significant correlation between the prevalence of oxidative damage-associated mutations with *in vivo* Mtb mutation rates in SIV + ART + TB animals, but not for the TB-only or SIV + TB animals (Fig. 2I). One hypothesis could be that Mtb is experiencing a range of oxidative damage in the SIV + ART + TB animals, and this can lead to increased Mtb mutation rates in a subset of animals. If this oxidative damage is host-directed, this may be recorded as earlier appearance of mutations at origin sites; consistent with this, we find that lung granulomas with intrahost mutations were detected significantly earlier by PET-CT in the SIV + ART + TB animals compared to the TB-only group (Fig. S1E). Together, the data suggest that despite ART viral suppression leading to similar bacterial outcomes (Fig. 2A through C), the Mtb intrahost mutational trajectory in these animals may be distinct from non-SIV animals, instead favoring a pattern of increased oxidative-associated damage.

### Intrahost mutations uncover dissemination events within barcoded Mtb networks

By integrating our whole-genome sequencing with previous barcoding data, we were able to link 79 intrahost mutations with the Mtb-barcoded strain that harbors these variants (see Materials and Methods). Of these, 67 mutations were found in a single tissue (considering all sampled lung, lymph node, and extrapulmonary sites), while 13 variants were shared across tissues (Fig. 3), suggesting they likely arose once and were spread through subsequent dissemination events. As only some tissues colonized by the same barcoded Mtb strain may harbor a given intrahost mutation, we used *in vivo* variants as additional “barcodes” with which to infer the order of dissemination events in each Mtb dissemination network. As an annotated example, we identified four *in vivo* variants occurring in the same Mtb-barcoded strain (Q25_GCTCAGTC) background in NHP 23218 (Fig. 3, bottom right). By mapping the intrahost variants present and absent in these tissues, we are able to infer at least three distinct dissemination events: first, two tissues represent dissemination of the wild-type parent strain; second, a TCCCAC>T deletion at position 182022 (red ring) emerged and was then disseminated to 12 tissues; and third, a new 277532_C>T variant (black ring) arose on top of the TCCCAC>T deletion, and both mutations were then disseminated to seven other sites. Finally, two variants—2554553_C>T (orange ring) and 2806057_G>T (blue ring)—emerged independently in single tissues, likely after prior dissemination events.

Similar branched networks were inferable across other animals (e.g., NHP 22517 [gray], NHP 33519 [green], and NHP 22117 [yellow]), supporting a model where Mtb dissemination is structured as parallel subnetworks of dissemination rather than a single radiation event. Further, we find seven instances (Fig. 3, arrows) where we can infer directional dissemination (i.e., where a mutation appears unfixed at an origin site and then hitchhikes as a fixed mutation to downstream tissues). Of these, five dissemination events involve Mtb spreading from and to lung sites (Fig. 3, red arrows), while two cases involve dissemination to lung sites from a thoracic lymph node (Fig. 3, blue arrows), suggesting that Mtb can, in principle, return to the lung after dissemination to other anatomical sites.

### Mtb mutations in NHPs and humans are enriched in lipid metabolism and biosynthetic processes

**Fig 4:**
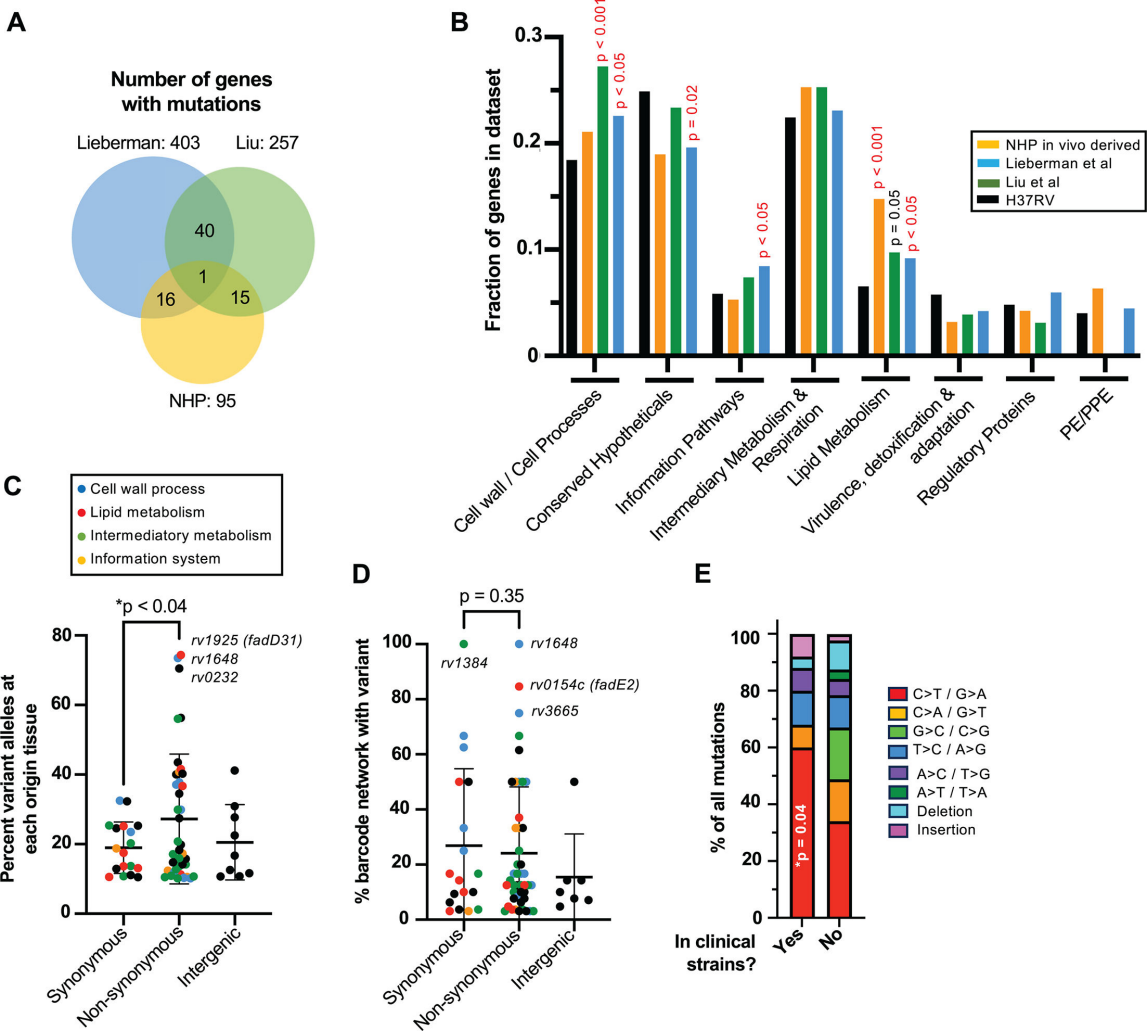
Intrahost mutations are enriched in lipid metabolism genes and confer Mtb fitness. (**A**) Overlap of genes with intrahost mutations (synonymous and non-synonymous) in NHPs (95 genes) and two human data sets (257 and 403 genes for Liu et al. (17) and Lieberman et al. (18), respectively). (**B**) The fraction of intrahost mutations in genes from each MycoBrowser category is plotted. The enrichment of intrahost mutations in each functional category was calculated against the number of genes annotated for each category in the H37Rv genome using Fisher’s exact test. (**C**) The proportion of mutant reads versus all reads (mutant and wild-type alleles) was compared at each origin tissue. (**D**) The percent of tissues containing a barcoded Mtb strain that also carried a genomic variant of interest was compared. (**C, D**) Statistical analyses were performed using an unpaired *t*-test. (**E**) Fisher’s exact test was used to compare the proportion of C-to-T transitions for variant alleles that were and were not found across 55,000 human clinical strain genomes.

We next correlated the NHP intrahost mutations with two treatment-naïve human studies that also identified intrahost Mtb mutations: Liu et al. (17) (321 mutations found in 257 genes in sputum from non-HIV-infected patients with active TB) and Lieberman et al. (18) (518 intrahost mutations in 403 genes from an autopsy study of HIV-coinfected individuals). In all three data sets, intrahost mutations were mostly found once in different genes, which precluded looking at the ratio of non-synonymous to synonymous mutations on a gene-by-gene basis; and only the iron transporter, *irtA,* was mutated in all three datasets (Fig. 4A). Despite the lack of one-to-one gene overlap, there was a significant enrichment of intrahost mutations in MycoBrowser (26)-predicted lipid metabolism genes (Fig. 4B) in the NHP data set (15% of mutations) and Lieberman et al. (9% of mutations) data set, with a statistical trend (*P* = 0.05) in the study by Liu et al. (10% of mutations).

Further, we find that intra-NHP mutated genes had significantly more predicted interactions than expected by chance (*P* = 0.005) using the STRING database (27) and that the putative gene interactions clustered by MycoBrowser functional categories, with lipid metabolism genes (especially *pks* genes) centrally located (Fig. S2). InterPro analysis identified several protein domains that were enriched in the NHP data set (Fig. S3), which included lipid metabolism domains (e.g., acyltransferase [FDR < 1 × 10^−5^]) and polyketide synthase-associated domains (FDR < 3 × 10^−3^). The genes associated with these terms were highly overlapping and included *pks7, pks8, pks12, mbtD, pks1,* and *pks2*, which are involved in the biosynthesis of diverse cell wall lipid structures and secondary metabolites. As in NHPs, we observed enrichment of polyketide domain terms in the human data sets as well (Fig. S3), suggesting these biosynthetic genes as a whole are common targets of mutation *in vivo*.

### Intrahost mutations are enriched within tissues and are associated with dissemination

We next assessed whether *in vivo-*derived mutations can confer intrahost fitness benefits during infection, focusing on mutations that change the protein sequence (missense and indels), which are more likely to impact gene function and Mtb fitness compared to synonymous mutations. To do this, we considered Mtb fitness across two anatomical scales: either locally within an individual tissue or systemically through dissemination to multiple sites. First, to assess individual tissue fitness, we compared the proportion of non-synonymous and synonymous mutations to their wild-type counterparts at origin sites, finding that the proportion of non-synonymous mutations was significantly higher than that of synonymous alleles (Fig. 4C), suggesting that a subset of these mutations may be locally beneficial for Mtb. Second, to study dissemination, we compared the fraction of tissues infected by a barcoded Mtb strain that also contained a non-synonymous or synonymous genomic variant, with the hypothesis that dissemination-promoting mutations will be enriched within disseminated networks. Here, we did not observe a statistical difference between synonymous and non-synonymous mutations, though there is a wide range of dissemination in both groups (Fig. 4D). Interestingly, mutations that were associated with highly disseminated Mtb strains are distinct from those that are enriched in individual tissues, which may suggest that additional factors beyond local fitness contribute to dissemination.

### NHP-derived Mtb mutations also arise in human clinical strains

We finally assessed whether intrahost NHP mutations can be found across human Mtb clinical strains. To do this, we leveraged a recent phylogenetic analysis that mapped over 55,000 Mtb clinical genomes to an ancestral genome (28) and looked for identical mutations as those observed in our NHP data set. We find 25 mutations (13 non-synonymous, 9 synonymous, and 3 intergenic) that were also present in at least one human Mtb clinical strain (Table 1). Most of these mutations appear in a single clinical isolate, but some mutations (e.g., *pks7* frameshift at codon 1174 and non-synonymous mutations in *rv0785*, *rv0987,* and *rv3594*) arose independently across different infections and were successfully transmitted between people (e.g., a missense mutation in *rnj*, a gene whose loss has been associated with drug tolerance (29), arose once but was subsequently shared across 10 individuals). We also find an intergenic mutation 25 nucleotides upstream of the ESX-1 regulator *whiB6,* which is an area of high mutational diversity across clinical strains (28). Finally, we show that intra-NHP mutations that match mutations in clinical strains are significantly enriched for C>T/G>A mutations (Fig. 4E), further supporting a model in which immune oxidative damage is a driver of Mtb diversification in the host.

**TABLE 1:** NHP intrahost mutations are also observed in human Mtb clinical strains^*a*^

Gene	DNA change	Protein change	Mutation	No. of events	No. of strains
rv0785	C>CG	NonSyn	Ala561fs	2	2
rv0987	A>G	NonSyn	Ser710fs	3	3
pks7	CG>C	NonSyn	Ala1174fs	A: 5	A: 21
				B: 2	B: 3
rv0075	G>A	NonSyn	Gly175Ser	1	1
rv0943	G>A	NonSyn	Ala54Val	1	1
rv1648	A>G	NonSyn	Ser26Gly	1	3
pks8	C>T	NonSyn	Thr1274Met	1	1
lipJ	G>A	NonSyn	Ala390Val	1	4
rv2075	C>A	NonSyn	Val415Phe	1	1
rv2565	C>T	NonSyn	Ser292Phe	1	1
rnj	T>C	NonSyn	Arg361Gly	1	10
entC	C>T	NonSyn	Ala270Val	1	8
rv3594	G>A	NonSyn	Arg215His	2	3
fadD34	C>T	Syn	Gly143Gly	1	1
rv1520	T>C	Syn	Arg81Arg	1	1
rv1835c	C>T	Syn	Ala326Ala	2	2
rv2402	G>A	Syn	Ala324Ala	1	1
rv2426c	T>G	Syn	Ala256Ala	3	12
rv3228	G>A	Syn	Leu102Leu	2	6
fadE31	C>T	Syn	Asp190Asp	2	4
aftA	C>T	Syn	Arg5Arg	5	27
glfT2	G>A	Syn	Asn229Asn	1	3
pe-pgrs38-pbpB	C>A	IG	pe-pgrs38 (−20)	1	1
rv3268-rv3269	A>C	IG	rv3269 (−110)	1	2
whiB6-rv3863	C>T	IG	whiB6 (−25)	C>T: 4	C>T:4
				C>G:2	C>G:10

^*a*^ Identical genomic mutations in the NHP data set were matched to mutations observed in over 55,000 clinical isolates (28). Clinical strain mutations and the number of independent mutations events were predicted by comparing each clinical strain against an Mtb ancestral genome reconstruction. Nonsyn = non-synonymous, Syn = synonymous, and IG = intergenic. Pks7 mutations with an “A” and “B” indicate different mutations leading to a frameshift in the same codon. Two different types of intergenic whiB6 mutations were found at the same position.

## DISCUSSION

Defining the intrahost Mtb mutational repertoire within treatment-naive hosts may inform the evolutionary pressures shaping Mtb adaptation to the host and also record how immune environments may differ in individuals who have other comorbidities such as HIV coinfection. Here, sequencing of Mtb from infected NHP hosts finds that SIV coinfection was associated with significantly increased Mtb mutational diversity compared to Mtb-only animals. This appeared to be mainly driven by shorter generation times and greater overall Mtb replication capacity in SIV-infected hosts, which create more opportunities for mutations to occur. Conversely, increased oxidative immune pressure is not likely driving mutation, as chronic SIV coinfection is known to impair immune control in this NHP model (30, 31) and we observe fewer oxidative damage-associated mutations in SIV animals relative to Mtb-only controls, a finding that has also been reported in HIV-positive patients (17, 18).

Further, we also evaluated the impact of ART on Mtb evolution within SIV-infected hosts. Prior barcoding analyses suggested that SIV + ART + TB animals remained more permissive for Mtb dissemination to extrapulmonary sites, suggesting a level of continuing immune dysfunction despite virologic control (7). Here, we find Mtb from a majority of ART animals had an increased proportion of oxidative damage-associated mutations relative to TB-only animals and that this signal was positively correlated with higher overall mutational rates. We speculate that a subset of SIV + ART + TB animals could be mounting a dysregulated heightened inflammatory response to Mtb infection. Previous work has shown that alveolar macrophages from HIV-negative individuals on pre-exposure ART prophylaxis mount an altered immune response to subsequent Mtb exposure (32), and as we did not have samples from an SIV-negative ART-only group, we cannot discern if antiretroviral exposure alone or a combination of SIV and ART is responsible for this shift in Mtb oxidative damage. Beyond studying Mtb evolution during coinfection, we envision that structured population sequencing in combination with barcoding analyses can be leveraged in translational contexts such as in vaccine studies. For example, quantification of barcoded Mtb strains has found that intravenous BCG (IV-BCG) vaccination is associated with a long-lasting ability to restrict early Mtb bacterial establishment (33), and it would be interesting to use Mtb population sequencing to assess whether bacteria in IV-BCG-vaccinated hosts exhibit mutational profiles and trajectories reflective of enhanced immune pressure. Intrahost sequencing may also identify tissues enriched for Mtb mutation events that could be selected for deeper immune profiling and may aid the discovery of novel immune correlates of protection.

Our findings also contextualize bacterial evolution and dissemination within the host. First, as granulomas are typically colonized by a single bacterial founder and that different Mtb isogenic strains have heterogeneous dissemination capacity even within the same host (34), we were able to incorporate intrahost mutations as secondary “barcodes” to further study otherwise clonally barcoded strains and infer complex dissemination patterns where Mtb is likely spreading in parallel subnetworks originating from different tissues. This is in contrast to a single-event dissemination model, where all future disseminated sites are seeded from a single early lesion. We do note that a majority of the dissemination networks harbor mutations at a single tissue, which could be consistent with a single-event model, but given that there are few intrahost mutations detected in general, we cannot rule out parallel dissemination in these cases as well.

**Fig 5:**
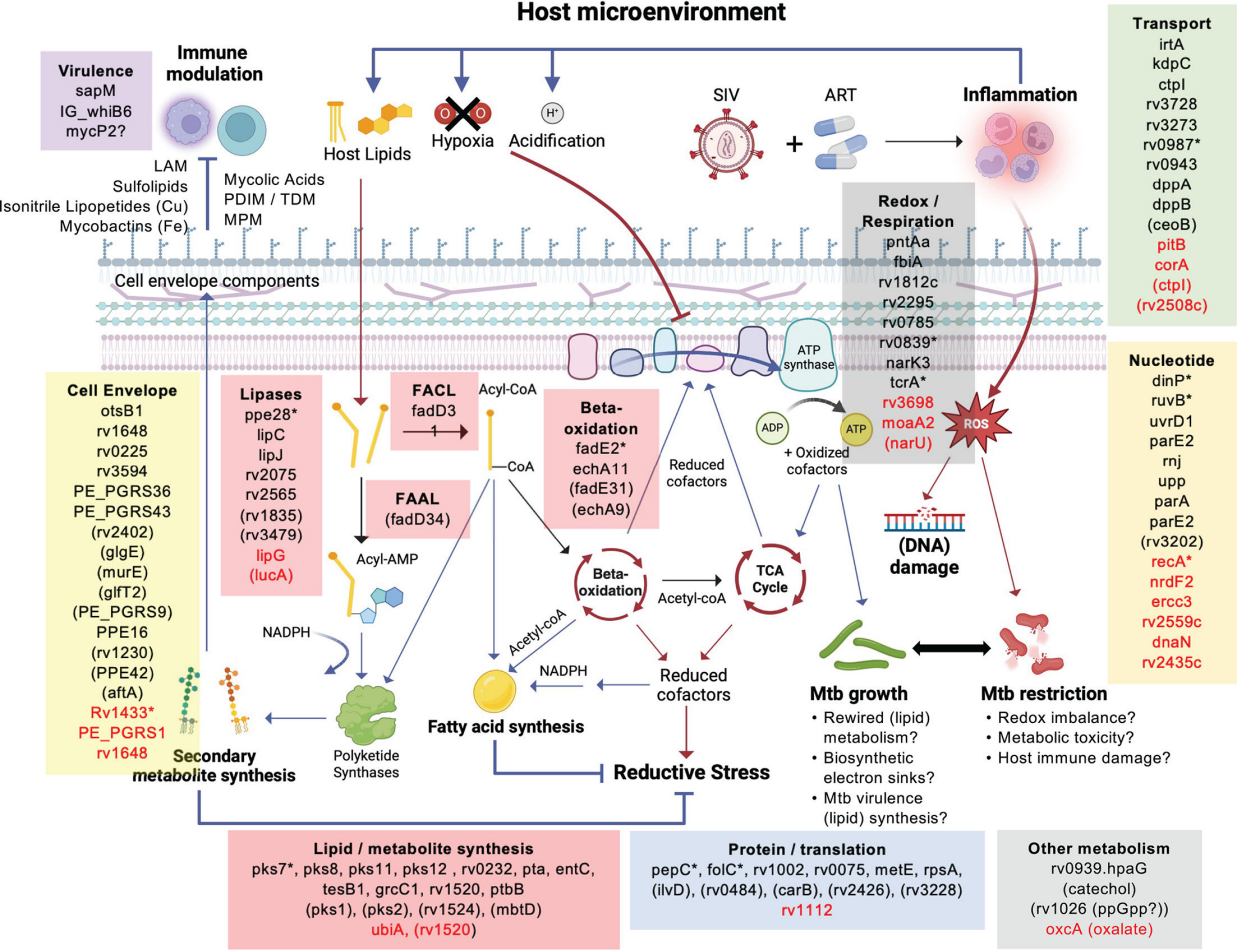
Model of intrahost mutations in metabolic and cell envelope processes. NHP-derived Mtb mutations are grouped into functional processes using literature curation and conserved domain similarity to microbial homologs. Genes in black represent all intrahost-derived Mtb mutations, while genes in red are mutations assigned to the “pre-existing” mutation group. Genes marked with asterisks represent frameshift mutations; genes in parenthesis represent synonymous mutations, and the remaining are predicted missense mutations. Functional categories are colored as follows: lipid metabolism and biosynthesis (red), cell envelope (yellow), nucleotide synthesis/repair (orange), metabolite transport (green), protein homeostasis/translation (blue), redox processes/respiration (gray), and virulence (purple). The schematic depicts lipases breaking down complex host and bacterial lipids into fatty acids, which can then be activated for degradation (or incorporated into other molecules) by conjugation to CoA through fatty acid CoA ligases (FACL). Different acyl-CoA substrates are then degraded through iterative cycles of beta-oxidation, yielding acetyl-CoA that can be used to drive the TCA cycle and respiration as well as being incorporated into Mtb secondary metabolites. Fatty acid ligation to AMP via fatty acid AMP ligases (FAAL) can be directly incorporated by pks enzymes (and other biosynthesis genes) into larger lipid-based structures, many of which are components of the cell envelope. Beta-oxidation of lipids also yields NADH, leading to increased reductive stress that needs to be re-balanced. This can occur through conversion of NADH to NADPH, which is utilized for secondary metabolite synthesis, as well as NADH consumption during respiration, which can become impaired due to host pressures like hypoxia, acidification, and inflammation. This may lead to leakage of electrons from the respiratory chain, generating reactive oxygen species and macromolecule damage in Mtb. In the context of some ART treated hosts, we hypothesize an augmented inflammatory environment not seen in Mtb-only animals, which may yield greater Mtb damage and increased detection of oxidative-damage associated mutations. The image was prepared using BioRender.

Second, our genomic mapping found that intrahost mutations were enriched in Mtb lipid catabolism and biosynthetic genes (Fig. 5), which may reflect the importance of beta-oxidation of host lipids for Mtb nutrient acquisition during infection (35). While beta-oxidation supports carbon acquisition, it also increases reductive stress by generating NADH, which can be counteracted through several mechanisms. First, NADH is consumed through respiration, and we observe mutations in putative nitrite efflux proteins *narK3* and *narU*, which may facilitate the use of nitrate as a terminal electron acceptor in the context of microaerophilic conditions (36). Second, NADPH equivalents are utilized for biosynthesis of cell envelope components and lipid storage molecules by large modular polyketide synthases (pks). The pks enzymes are responsible for producing key cell wall components (e.g., dimycocerosates, mycocerosic acids, lipoarabinomannan, mannosyl-ß−1 phosphomycoketides, sulfolipids, and the siderophore mycobactin) using lipid precursors (37); and recent work has shown that phthiocerol dimycocerosate production is required for optimal Mtb growth on propionate, which presumably acts to re-balance the reductive stress from odd-chain lipid degradation (38). Here, we find significant enrichment of intrahost mutations—both in NHP and human data sets—within *pks* genes, which may be beneficial to Mtb in the human population; for example, we identified a frameshift in *pks7* that also appears to be independently mutated seven times across human clinical strains and has been transmitted between 24 individuals (Table 1). Interestingly, while *pks7* knockout strains have been reported to be attenuated in aerosol infection in C57BL/6 mice (39), our data would suggest that this enzyme can be dispensable in human infection and transmission, which would be consistent with the findings of a recent work showing that *pks7* insertion mutants retain the ability to intravenously infect a genetically diverse panel of mice (40). Together, these mutational signatures highlight the metabolic flexibility of Mtb in balancing energy and redox needs under host-imposed stresses.

### Limitations to the study

Several limitations to this study remain. First, our data set likely underestimates true intrahost mutational diversity, as late-emerging mutations (which will be at lower prevalence compared to early arising mutations) are unlikely to be sampled for the sequencing depth used in this study. Further, some variants will also have been filtered out by our 10% prevalence threshold, which was imposed to reduce false positive calling of low-frequency sequencing errors. Finally, highly repetitive genes of the PE/PPE families are difficult to uniquely map using short-read sequencing technology, so we have masked many of these regions in our reference genome (20) for subsequent analyses.

Second, our analyses are restricted to an early infection time point when the bacterial population size remains sizeable but mutational diversity may be limited compared to human infection with Mtb, where infections of months to years enable Mtb to accumulate intrahost mutations. On the opposite end, human longitudinal studies have shown that antibiotic treatment (and presumably immune attack leading to latent infection) will shrink the *in vivo* airway mutant pool (13–15), which will likely eliminate subsets of mutations that were beneficial early—but not late—in infection. Further, this NHP study does not capture population bottlenecks occurring during inter-host transmission, where mutations that may be beneficial within a host also must successfully access the airway, survive exposure to the outside environment, and transmit infection to a new host. This high bar for spread between hosts may manifest as strong transmission bottlenecks and explain why the vast majority of mutations found across over 55,000 Mtb clinical strains exist at terminal branches within single individuals (28).

Finally, while gene-level data suggest ongoing Mtb mutation in lipid metabolism genes, it is difficult to infer whether a given missense or synonymous mutation is positively or negatively impacting gene function and thereby allow us to nominate pathways as essential or non-essential for host adaptation. Additionally, as the exact lipid substrates of many paralogs involved in lipid metabolism and biosynthesis remain unknown, future work will be needed to define the nutrient diversity in host microenvironments—especially at relatively inaccessible anatomic sites beyond sputum—that promote and restrict successful Mtb infection.

## MATERIALS AND METHODS

### Sequencing of Mtb genomic populations from infected NHP tissues

We curated a set of Mtb genomic DNA samples that were previously generated for barcode sequencing (7). In order to compare Mtb evolution across similar infection time scales, we chose to analyze samples from only the 20 NHPs from the original study that survived 10–12 weeks of Mtb infection, though we did sequence tissues from three additional SIV-coinfected NHPs that reached a humane endpoint much earlier. Whole-genome sequencing was performed on the Illumina NovaSeq S4 300 cycle platform across four sequencing runs (see Table S1 for sample metadata and sequencing accession codes). Reads were filtered for good sequencing-quality reads using fastp (41) and then mapped to the recently generated barcoded Mtb Erdman reference genome (GenBank accession number CP172229.1) (20), which was obtained from an aliquot of the library used to infect NHPs across studies. After alignment, we applied Mutect2 from the GATK toolkit (https://gatk.broadinstitute.org/hc/en-us) to identify all mutational changes (single-nucleotide polymorphisms and insertion/deletions) at any frequency relative to the reference genome. The functional effect of each mutation (e.g., intergenic, synonymous, missense, and frameshift) was also predicted using SnpEff based on our recent Erdman annotation (20). We included a mask to redact variants predicted in repetitive sites in our Erdman reference genome that had been predicted to have low mappability (20), as they are more likely to arise from mismapping events.

The Mutect2 output yielded a preliminary list of over 100,000 detected genomic changes across all samples, which were mainly found at low prevalence (<10% mutant alleles versus wildtype sequences) and driven primarily by a subset of samples. Due to the logistics of tissue sampling, each infected tissue was homogenized for Mtb plating, and the entire Mtb population was collected for gDNA purification. Therefore, we lack technical replicates from the same sample to assess the impact of tissue homogenization and bacterial gDNA extraction on introducing DNA mutations. However, we aimed to filter out the vast majority of potential artifactual sequencing errors (though false positives may still remain) by enacting three QC steps: first, we used the interquartile range (IQR) method to define outlier samples with a number of Mutect2 variants at 1.5× greater than the IQR across all the samples from each sequencing batch. This yielded 46 outlier samples that were removed from subsequent analyses (Fig. S1A, “QC1”). Notably, the vast majority of mutations in these outliers were enriched for C-to-A transversions, a mutational change that has been previously associated with DNA damage artifacts arising during library preparation due to elevated heat (23). Second, we filtered the remaining variants to remove low-confidence calls at sites with substandard sequencing depth, which included only (i) sequencing runs with an average read depth above 50×; (ii) genomic variants that sat in regions where there was overall sequencing depth that exceeded 50% of the average sequencing depth of the sample; and (iii) variants whose prevalence was above 10% (relative to all reads at that position) to account for rare sequencing errors (Fig. S1A, “QC2”). Given the stochastic nature of DNA damage during library preparation, which should yield rare mutations, we also expect that the minimum prevalence threshold will aid in removal of library preparation artifacts. Third, due to the difficulty in uniquely assigning short reads to repetitive regions in the Mtb genome, we reasoned that there will be systematic mismapping events yielding the same exact “mutation” (in both chromosomal position and type of DNA base change) being found in samples even across different animals. Filtering out the exact same variants found in more than one animal yielded a candidate list of 217 intrahost variants found across 125 tissues (Fig. S1A, “QC3 step”). Finally, we visually inspected the read pileups generated by each mapping file using Samtools and confirmed that the different reads were mapping across the site of interest and that variants were not clustering at the 5′ and 3′ ends of reads. This produced our final validated table of 143 unique high-confidence *de novo* mutations for further analysis (Table S2).

### Defining pre-existing and intrahost-derived Mtb mutations

As our reference Erdman genome was derived from a single barcoded clone within a larger barcoded library, we cannot exclude that some mutations identified by sequencing could have arisen *in vitro* and is specific to a given barcoded strain. To conservatively separate *in vivo-*derived mutations versus those that may have been pre-existing in the library, we compiled the Mtb-barcoded strains present in each tissue sample from prior barcode amplicon sequencing (7) and compared this to the Mutect2-detected variants present in each tissue. As diagrammed in Fig. 1B, we considered mutations *in vivo*-derived if they satisfied one of the two following criteria.

First, we first looked for tissues that contained a genomic variant and a single barcoded Mtb strain, which allowed us to link a genomic variant to a barcoded Mtb strain genome. We then identified all tissues containing that barcoded Mtb strain and assessed the presence/absence of the genomic variant of interest in those tissues. If we observed a pattern where some tissues had only the wild-type allele while others had the genomic variant, we reasoned the detected mutation likely originated during infection. This situation is diagrammed as “BC2” in Fig. 1B.

Second, when we cannot link a genomic variant to a single barcoded Mtb strain (often the case when a mutation is found in a tissue that was infected by more than one barcoded Mtb strain), we looked for the genomic variant of interest across all tissues containing every possible barcoded Mtb strain it could be linked to. If we observed both presence of wild-type Mtb and mutant alleles in every potential barcode network, we also considered this variant as likely emerging *in vivo*. This situation is diagramed as “BC3” and “BC4” in Fig. 1B.

If a variant does not satisfy the criteria above, we cannot rule out that the mutation could have emerged *in vitro*, so it is considered “pre-existing” and excluded from further analyses. An example of this is “BC1” in Fig. 1B, where a mutation was present in all tissues that were infected in that barcoded strain.

### Defining mutant origin tissues and timing of origin emergence

To define “origin” sites of intrahost mutations, we first identified tissues containing a single barcoded Mtb strain. If sites contain an unfixed mutant allele (i.e., <75% of all reads at that site are mutant), then we considered these tissues as harboring both wild-type (i.e., parental) and mutant alleles. These are likely the origin sites where the mutation originally arose. While we elected to use a 75% threshold based on the WHO threshold used to define high-confidence mutations associated with drug resistance (42), we also empirically find that using a 90% threshold would not have changed the origin site designations in this study. In the parent study, animal lungs were imaged using PET-CT on a monthly basis at 4, 8, and 12 weeks post-infection to register the location and presence of newly detected lung lesions (7). Here, we compared the time of PET-CT detection of the lung-specific origin sites (PET-CT registration was not done for thoracic lymph node and extrapulmonary sites) across treatment groups.

### Bacterial burden quantification and flow cytometry analysis of lesions across animals

We obtained previously published tissue-level live bacterial burden (CFU) and total (live and dead) bacterial chromosomal equivalent (CEQ) measurements for the animals that were included in this study (7). Total CFU and CEQs were summed for all sampled tissues from each animal for statistical comparisons. We also obtained lesion-level flow cytometry profiling data from the prior study (7), in which CD11b-positive myeloid cells were profiled for the expression of activation and cytokine markers.

### Mutation rate quantification

Similar to previous work using colony-based sequencing from infected NHPs (16), we calculated an Mtb mutation rate for each animal by dividing the number of unique mutational events seen (after normalizing for the number of tissues sampled) by the number of bacterial generations (per unit of infection time) occurring in the host. Here, we summed the number of different mutations across all tissues in an NHP to estimate the mutation rate of the entire Mtb population within one animal using the following equation:


μ=m/[N×(t/g)]/D

In this equation, μ represents the total mutation rate across an animal, *m* is the number of unique mutational events detected by sequencing across all tissues in the animal, *N* is the Mtb genome size (set at 4,400,000 bp), *t* is the total infection time of each NHP (days of infection multiplied by 24 h), *g* is the Mtb *in vivo* generation time (in hours), and *D* is the total number of tissues that were sequenced per animal.

We applied two versions of this calculation. First, to establish a relative mutation frequency per day of infection (Fig. 2A), we used a fixed 18 h for a single doubling across all treatment groups. The numerical values calculated with a fixed doubling time are the relative mutation frequencies per fixed unit of infection time, which was used to establish a fold change difference between treatment conditions (relative to the mean mutation frequency in the Mtb-only group). Second, to calculate a specific Mtb mutation rate per generation (Fig. 2D), we first estimated the average Mtb generation time (*g*) in each NHP using the following formula:


g=t/(log2 (final Mtb population size/founding Mtb population size)).

In this equation, *t* is the total infection time of each NHP as described above, while we used the total number of Mtb CEQs obtained across all infected tissues in each animal as the final Mtb population size and the number of unique barcodes found in each NHP as the starting Mtb founder population size. We then used each NHP-specific generation time (*g*) to calculate the Mtb mutation rate for each animal using the first equation.

### Bacterial barcoding network visualization

Using previously published amplicon barcoding data (7) from the tissues sequenced in this study, we generated network plots by converting a table of genomic variants present in all sequenced tissues into a network plot using the R package, igraph. Specifically, each tissue that contained a given barcoded Mtb strain was initially plotted as nodes given the same color. A ring was added in a different color to a tissue/node if it contained a unique genomic variant after sequencing. Different variants within the same barcoded dissemination network are represented by different colored rings. Using the presence or absence of genomic mutations shared across tissues, we inferred dissemination subnetworks with the fewest dissemination events. In the case of networks for which we are unable to order the events of dissemination, we arbitrarily represented dissemination as a single star-like network. If a genomic variant was found in more than one tissue with a network, we manually re-ordered connecting lines between these nodes to form subnetworks. We finally imported the igraph barcode networks into Adobe Illustrator to visually improve line definition, update colors for visualization, and scale for size. The edgelist representing the connections between tissues for all visualized barcoded networks is provided as an R object in the data folder on the Github page: https://github.com/Fortune-Lab/Mtb-NHP-genotyping. Note: some tissues harbored multiple genomic mutations, but igraph is limited to adding a single ring, so we added these variants and the site of origin sites (as boxes) in Adobe Illustrator. The presence of all genomic mutations and origin sites within the networks is indicated by metadata columns in the R edgelist object.

### STRING and InterPro analyses

The list of all genes with *in vivo-*derived mutations from our NHP data, Liu et al. (17), and Lieberman et al. (18), were analyzed on the STRING database (https://string-db.org) to define putative interactions. All interacting nodes were then colored by their predicted Mycobrowser (26) functional categories. Finally, protein domain enrichment analysis (InterPro) was performed on the STRING database for all genes.

### Identification of intra-NHP mutations in human Mtb clinical strains

We obtained the list of genomic mutations identified across approximately 55,000 publicly available Mtb clinical isolate sequences from a recent publication (28), which mapped clinical strains against a reconstructed ancestral Mtb genome and constructed a phylogenetic tree to identify independent mutational events and sharing of mutations across individuals. We searched for NHP-derived intragenic mutations that also showed up with the same DNA mutation (e.g., C>T) in the same codon position in the human Mtb clinical isolate data. For intergenic sequences, we used BLAST to search for identical stretches of the genomic sequence around the mutation site and then identified the relevant genomic position of the mutation in the ancestral Mtb genome (based on H37Rv positional coordinates) used to map the human clinical isolates.

## Supplementary Material

Reviewer comments

## Data Availability

The genomic mapping, quality control, and variant detection code and methods are fully described at the following Github link: https://github.com/Fortune-Lab/Mtb-NHP-genotyping. All sequencing data have been deposited on the Sequencing Read Archive, with accession codes for individual samples provided in Table S1. All sequencing data are also available under BioProject accession number PRJNA1432747. All metadata associated with the data sets in this work are deposited on FAIRDOMHub at the following link: https://fairdomhub.org/studies/1401.

## SUPPLEMENTAL MATERIAL

Fig. S1 msystems.00329-26-s0001.pdf Genomic variants detected by Mutect2, Mtb barcode establishment, *in vivo* generation time, and PET-CT detection time.

Fig. S2 msystems.00329-26-s0002.pdf STRING analyses showing the predicted interactions between all genes in the NHP data set.

Fig. S3 msystems.00329-26-s0003.pdf All genes with intrahost mutations for each data set were analyzed for InterPro protein domain enrichment on the STRING database.

Table S1 msystems.00329-26-s0004.xlsx Genomic accession numbers for all sequenced Mtb populations from infected tissues in this study.

Table S2 msystems.00329-26-s0005.xlsx High-confidence Mutect2-detected genomic mutations occurring *in vivo*.

OPEN PEER REVIEW reviewer-comments.pdf An accounting of the reviewer comments and feedback.

ASM does not own the copyrights to Supplemental Material that may be linked to, or accessed through, an article. The authors have granted ASM a non-exclusive, world-wide license to publish the Supplemental Material files. Please contact the corresponding author directly for reuse.
