# Supplementary material for: Population-level genome sequencing reveals distinct Mycobacterium tuberculosis intrahost mutational trajectories in simian immunodeficiency virus-coinfected and antiretroviral treated non-human primates
Source: mSystems. 2026 Jul 27;11(8):e00329-26. doi: 10.1128/msystems.00329-26 (PMC13487684; doi:10.1128/msystems.00329-26)

Supplementary Figure 3. All genes with intrahost mutations (all mutations = synonymous and non-synonymous mutations) for each dataset was analyzed for InterPro protein domain enrichment on the STRING database.

NHPs (all mutations)

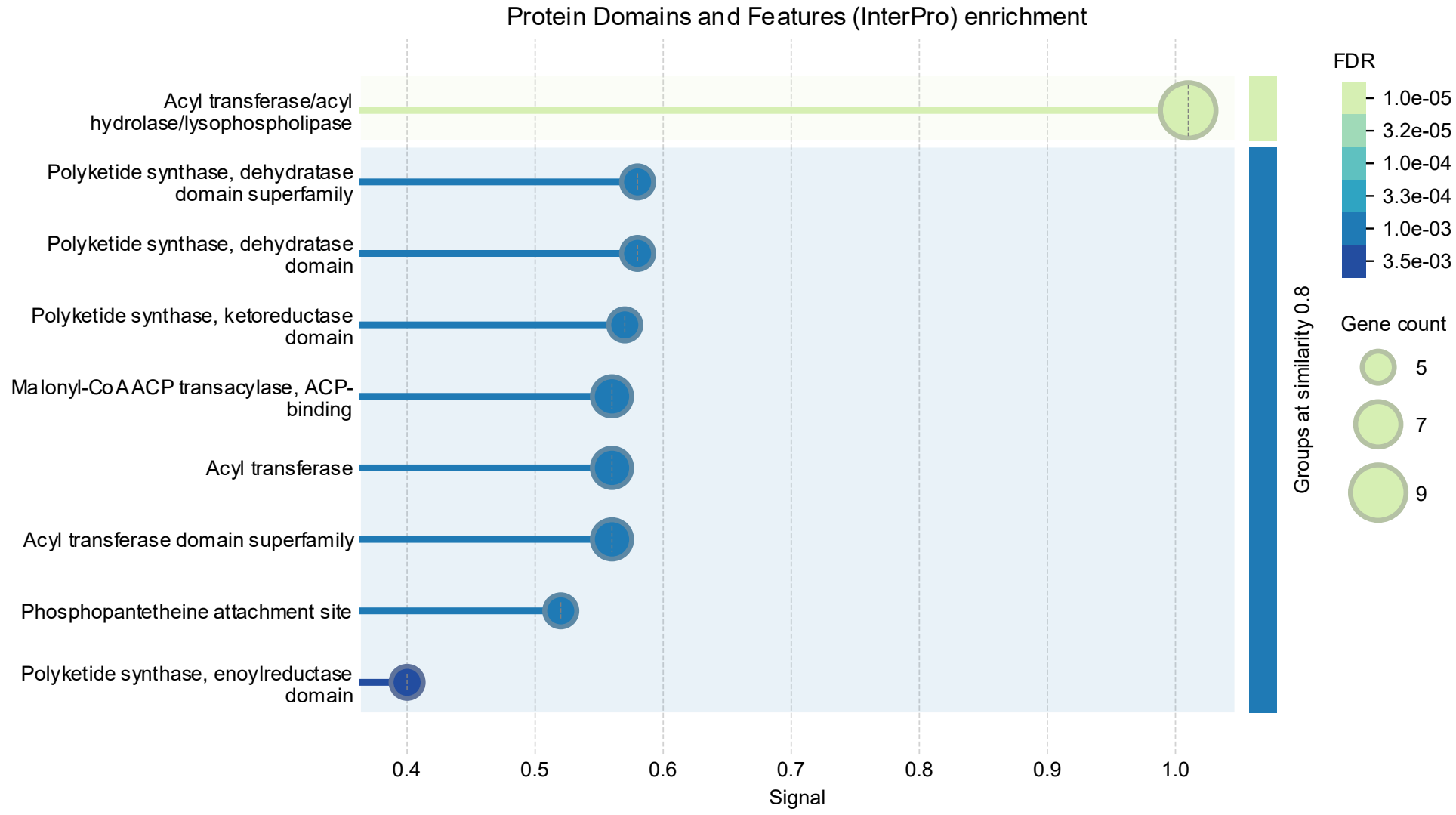

Liu et al. (all mutations)

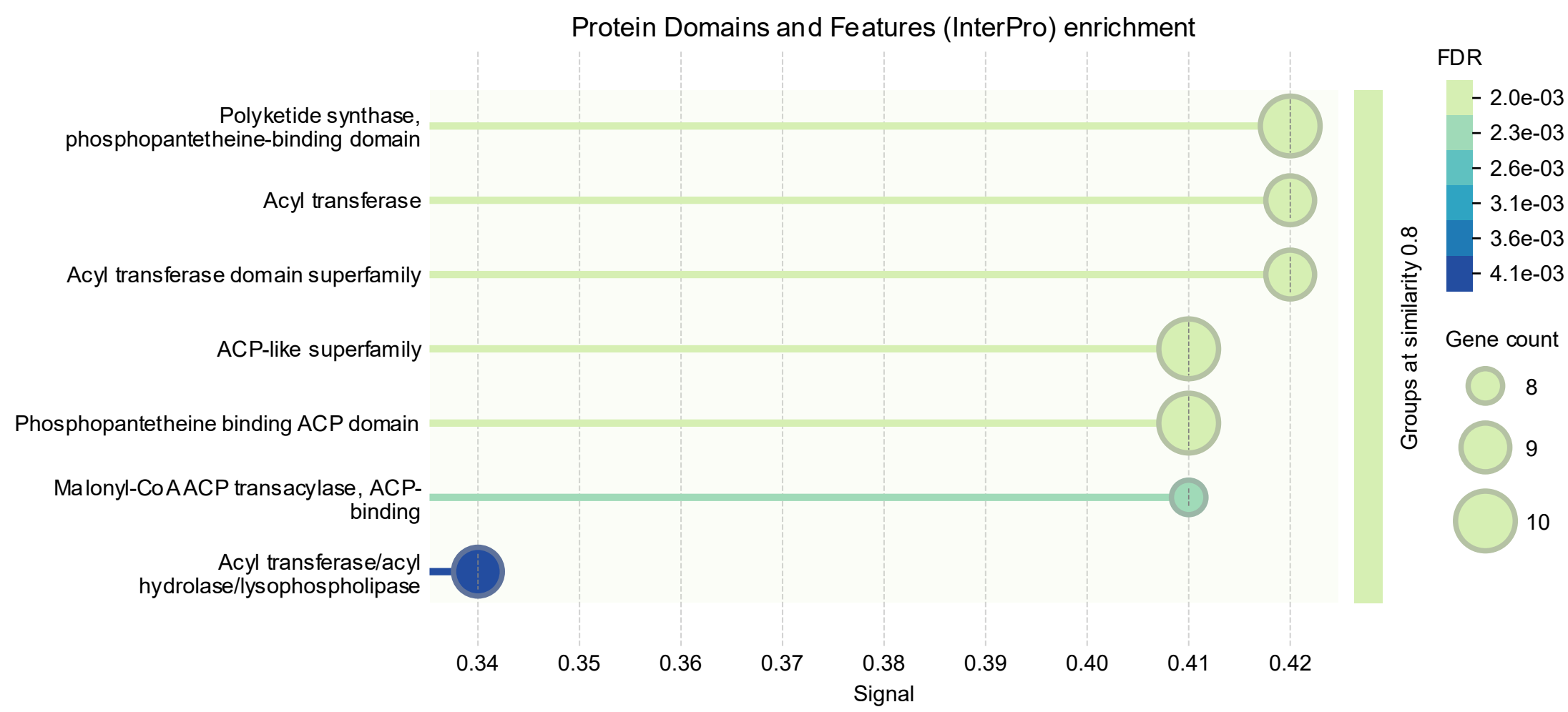

Lieberman et al. (non-synonymous mutations)

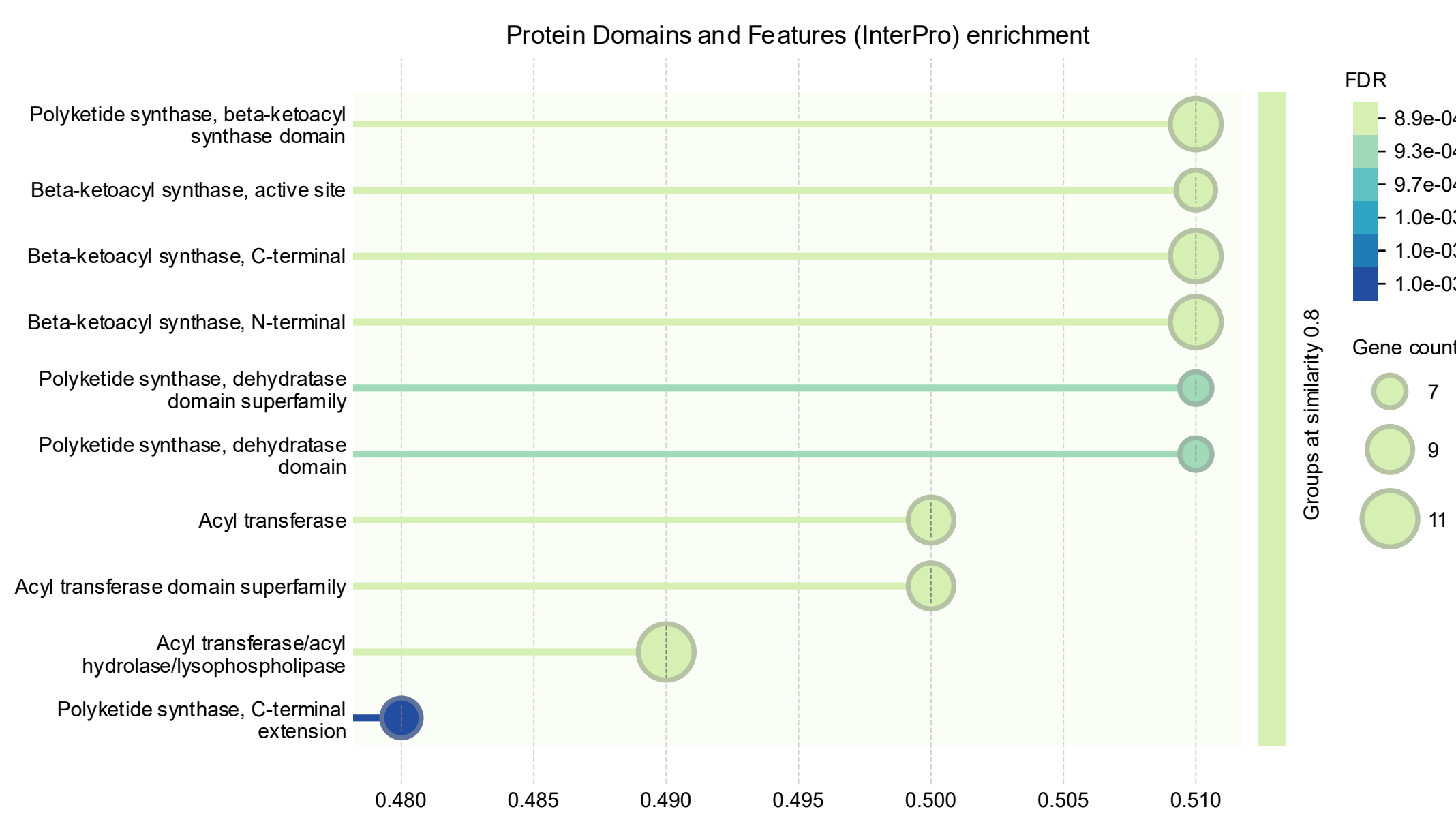

Supplement: Supplementary file 3 [file msystems.00329-26-s0003.pdf]
