# Supplementary material for: Population-level genome sequencing reveals distinct Mycobacterium tuberculosis intrahost mutational trajectories in simian immunodeficiency virus-coinfected and antiretroviral treated non-human primates
Source: mSystems. 2026 Jul 27;11(8):e00329-26. doi: 10.1128/msystems.00329-26 (PMC13487684; doi:10.1128/msystems.00329-26)
Supplement: Supplementary file 4 [file reviewer-comments.pdf]

AMERICAN SOCIETY FOR MICROBIOLOGY | mSystems®

Population-level genome sequencing reveals distinct Mycobacterium tuberculosis intrahost mutational trajectories in simian immunodeficiency virus co-infected and antiretroviral treated non-human primates

Michael Chao, Michael Chase, Shoko Wakabayashi, Andrew Vickers, Byron Roman, Forrest Hopkins, Peter Culviner, Maximilian Marin, Pauline Maiello, Collin Diedrich, Zandrea Ambrose, Philana Lin, Qingyun Liu, and Sarah Fortune

Corresponding Author(s): Michael Chao, Harvard TH Chan School of Public Health

Review Timeline:		
	Submission Date:	March 30, 2026
	Editorial Decision:	May 7, 2026
	Revision Received:	June 4, 2026
	Accepted:	July 6, 2026

Editor: Min Yue

Reviewer(s): Disclosure of reviewer identity is with reference to reviewer comments included in decision letter(s). The following individuals involved in review of your submission have agreed to reveal their identity: Guangzhi Zhang (Reviewer #2)

Transaction Report:

(Note: With the exception of the correction of typographical or spelling errors that could be a source of ambiguity, letters and reports are not edited. The original formatting of letters and referee reports may not be reflected in this compilation.)

DOI: https://doi.org/10.1128/msystems.00329-26

Re: mSystems00329-26 (Population-level genome sequencing reveals distinct Mycobacterium tuberculosis intrahost mutational trajectories in simian immunodeficiency virus co-infected and antiretroviral treated non-human primates)

Dear Dr. Michael C Chao:

Thank you for the privilege of reviewing your work. Below you will find my comments, instructions from the mSystems editorial office, and the reviewer comments.

As you see, we collect all the comments from three individual reviewers. Please provide further improvements, as per reviewers' comments.

Please return the manuscript within 60 days; if you cannot complete the modification within this time period, please contact me. If you do not wish to modify the manuscript and prefer to submit it to another journal, notify me immediately so that the manuscript may be formally withdrawn from consideration by mSystems.

Revision Guidelines
To submit your modified manuscript, log into the submission site at https://msystems.msubmit.net/cgi-bin/main.plex. Go to Author Tasks and click the appropriate manuscript title to begin. The information you entered when you first submitted the paper will be displayed; update this as necessary. Note the following requirements:

• Upload point-by-point responses to the issues raised by the reviewers in a file named "Response to Reviewers," NOT in your cover letter.
• Upload a compare copy of the manuscript (without figures) as a "Marked-Up Manuscript" file.
• Upload a clean .DOC/.DOCX version of the revised manuscript and remove the previous version.
• Each figure must be uploaded as a separate, editable, high-resolution file (TIFF or EPS preferred), and any multipanel figures must be assembled into one file.
• Any supplemental material intended for posting by ASM should be uploaded with their legends separate from the main manuscript. You can combine all supplemental material into one file (preferred) or split it into a maximum of 10 files with all associated legends included.

For complete guidelines on revision requirements, see our Submission and Review Process webpage. Submission of a paper that does not conform to guidelines may delay acceptance of your manuscript.

Data availability: ASM policy requires that data be available to the public upon online posting of the article, so please verify all links to sequence records, if present, and make sure that each number retrieves the full record of the data. If a new accession number is not linked or a link is broken, provide mSystems production staff with the correct URL for the record. If the accession numbers for new data are not publicly accessible before the expected online posting of the article, publication may be delayed; please contact production staff (mSystems@asmusa.org) immediately with the expected release date.

Publication Fees: For information on publication fees and which article types are subject to charges, visit our website. If your manuscript is accepted for publication and any fees apply, you will be contacted separately about payment during the production process; please follow the instructions in that e-mail. Arrangements for payment must be made before your article is published.

ASM Membership: Corresponding authors may join or renew ASM membership to obtain discounts on publication fees. Need to upgrade your membership level? Please contact Customer Service at Service@asmusa.org.

The ASM Journals program strives for constant improvement in our submission and publication process. Please tell us how we can improve your experience by taking this quick Author Survey.

Thank you for submitting your paper to mSystems.

Sincerely,
Min Yue
Editor
mSystems

Reviewer #1 (Comments for the Author):

This is an outstanding and technically sophisticated study that leverages population-level whole genome sequencing to interrogate Mycobacterium tuberculosis (Mtb) evolution within non-human primate hosts. By analyzing approximately 480 individual tissue lesions across animals with distinct immune contexts-including SIV coinfection and antiretroviral therapy-the

authors achieve an exceptional level of spatial and population resolution. The work provides compelling evidence that Mtb undergoes dynamic and structured intrahost diversification, identifies both tissue-restricted and disseminated mutational events, and reveals how immune perturbation reshapes mutational trajectories. The identification of recurrent mutations, including those overlapping with human clinical isolates, further underscores the biological and translational relevance of the findings. In the spirit of Pasteur's observation that scientific progress continually opens new fields of inquiry, this work expands our view of Mtb evolution within the host. Overall, this study represents a tour de force in the analysis of pathogen evolution in vivo and addresses a long-standing question in the field regarding the extent and significance of Mtb diversification.

Minor points for improvement

Estimation of population size underlying SNP emergence
1. The population-level sequencing approach is a major strength of the study, and it raises an interesting quantitative question. Is it possible to estimate, even approximately, the effective bacterial population sizes within individual lesions or tissues from which the observed SNPs arise? Such estimates-whether derived from variant frequencies, mutation rates, or modeling approaches-would provide valuable context for interpreting the likelihood of mutation emergence, the balance between selection and drift, and the observed differences across experimental groups.
2. Implications for TB vaccination studiesGiven the power of this approach to resolve intrahost evolutionary dynamics, it would be valuable for the authors to comment on how similar population-level sequencing strategies could be applied in future studies of TB vaccination. For example, what expectations might one have regarding mutational trajectories, bottlenecks, or selective pressures in vaccinated versus unvaccinated hosts? A brief discussion of how this framework could inform evaluation of vaccine-induced immune pressures would broaden the impact and translational relevance of the work.

Reviewer #2 (Comments for the Author):

Overall Assessment:
This study employs a non-human primate (NHP) model with SIV co-infection and ART treatment to conduct large-scale whole-genome sequencing of Mycobacterium tuberculosis (Mtb) isolated from approximately 480 infected tissues. A total of 116 intra-host mutations were identified, and the mutation rates, mutational profiles, and enrichment of mutations in lipid metabolism-related genes under different immune conditions were systematically analyzed. The key strengths of this work include the comprehensive characterization of Mtb intra-host mutational landscapes utilizing multi-tissue sampling in NHP models, and the innovative application of intra-host mutations as secondary barcodes to trace bacterial dissemination in vivo. The findings provide valuable insights for understanding Mtb adaptive evolution under HIV-TB co-infection. The manuscript is well organized and structurally sound; however, several critical aspects require further discussion, clarification, and revision.

Major comments:
1.The limitations of relative mutation rate calculation need to be thoroughly addressed. The authors adopted a fixed generation time of 18 hours for mutation rate estimation and acknowledged that the actual in vivo generation time remains unknown. The elevated mutation rate in the SIV group (Fig. 2A) is attributed to enhanced mutation induced by immune suppression, which may be misleading. This difference could alternatively arise from accelerated bacterial replication and shorter generation time in the SIV group. The marginal trend in CEQ values (p=0.09, Fig. 2B) and elevated CFU levels (Fig. 2C) only reflect increased bacterial load, rather than accelerated replication rates. Higher CFU may result from impaired host clearance, as noted by the authors. It is recommended that the authors explicitly discuss the potential bias introduced by the fixed generation time assumption, perform sensitivity analyses to validate the robustness of conclusions under different generation time scenarios, and adopt more cautious interpretations regarding the correlation between immune suppression and elevated mutation rates.

2.The causal relationship between oxidative stress-associated mutations and host immune pressure is insufficiently validated. The authors attribute C>T/G>A transitions to oxidative damage induced by host immune responses, supported by reduced bacterial load in mutation-origin tissues (Fig. 2D). However, several concerns remain. First, although C>A artifacts were excluded, potential oxidative damage introduced during tissue processing and DNA extraction has not been assessed, and appropriate technical replicates or control measures are lacking. Second, the increased proportion of oxidative mutations in the SIV+ART+TB group is attributed to dysregulated inflammation, without empirical evidence from immunological indicators, such as cytokine profiles, reactive oxygen species measurements, or macrophage activation markers. The authors should discuss the potential artifacts from sample processing, moderate their interpretations regarding ART-induced inflammatory stress, and explicitly state that the causal link between oxidative damage and mutational patterns remains hypothetical.

3.The interpretation of cross-species comparison requires further refinement. The authors compare NHP-derived mutations with a dataset of 55,000 human clinical isolates and highlight that 81% of detected mutations are absent in human strains. It should be noted that human clinical isolates represent globally disseminated lineages filtered by strong transmission bottlenecks. In contrast, most intra-host mutations identified in NHP are transient and not expected to be captured at the population level. Rather than emphasizing mutation overlap ratios, the discussion should focus on convergent mutations emerging independently in both human and NHP populations, which provide key evidence for adaptive convergence.

Minor comments:
1.Inconsistent descriptions of sample size exist: "approximately 480 infected tissues" in the abstract and "482 tissues" in the results. Uniform terminology is recommended throughout the manuscript.

2.The methodological description for Fig. 1C is incomplete. The grey shaded area represents the 95% confidence interval derived from 1,000 simulations; however, it remains unclear whether genomic features such as GC content, repetitive regions, and alignment complexity were adjusted for in the simulation procedures. Supplementary details are required.

3.The threshold for defining mutation origin tissues is not justified. Mutant allelic frequency below 75% is used to identify origin sites. The authors should provide literature support or explicit rationale for this threshold.

Reviewer #3 (Comments for the Author):

Chao and colleagues present a fascinating study identifying the intrahost mutational landscape of Mtb infected non-human primates in the context of Mtb-SIV coinfection and with antiretroviral therapy. The study encompasses a large sequencing study of hundreds of barcoded Mtb infected tissues across 20 NHPs under infection, confection or treatment conditions. From the total mutations identified that emerge during infection, the authors identify subsets of tissue specific mutations versus universal across tissues and across treatment groups. They show a higher mutation rate in SIV infected NHPs, concordant with increased bacterial outgrowth. Gene groups of interest include lipid metabolism and pks genes. Interestingly, the authors also identify NHP-derived mutations, that are independent from clinical isolated from human populations. Overall, this is a thorough and well conducted study that is very timely for the field in identifying early infection mutational events. Importantly, this works also sheds light on dissemination events and various immune pressure encountered during co-infection and treatment.

No major comments.

Minor comments:
-What was the proportion of male and female NHPs in the infection studies, including the new samples? Does sex have any effect on mutational rates across the samples? Curious if this is linked with immune pressures or worth commenting on in the discussion.
-Even though these are curated from a previous study, recommend the authors include the IACUC statement in methods (as this study includes several new samples)

First, we are grateful to all three reviewers for taking the time to evaluate our manuscript and for considering our results a useful contribution for the field. Second, we thank the reviewers for their suggestions, which we have used to strengthen our manuscript with expanded discussion and new analyses. Please see the 'marked up' text file to visualize the textual changes and a final 'clean' version to represent the current finalized manuscript. Line numbers refer to the marked-up version.

Below, we individually address the reviewer comments in red, which has broadly yielded the following structural changes:

- Updated Figure 1C to correct a plotting error
- Updated Figure 2A
- New Figures 2D and 2E to define in vivo Mtb replication and mutation rates
- New Supplementary Figures 1B-D to define Mtb populations and in vivo generation times
- Original Supplementary Figure 1C has now become Supplementary Figure 2.

Reviewer #1 (Comments for the Author):

This is an outstanding and technically sophisticated study that leverages population-level whole genome sequencing to interrogate Mycobacterium tuberculosis (Mtb) evolution within non-human primate hosts. By analyzing approximately 480 individual tissue lesions across animals with distinct immune contexts-including SIV coinfection and antiretroviral therapy-the authors achieve an exceptional level of spatial and population resolution. The work provides compelling evidence that Mtb undergoes dynamic and structured intrahost diversification, identifies both tissue-restricted and disseminated mutational events, and reveals how immune perturbation reshapes mutational trajectories. The identification of recurrent mutations, including those overlapping with human clinical isolates, further underscores the biological and translational relevance of the findings. In the spirit of Pasteur's observation that scientific progress continually opens new fields of inquiry, this work expands our view of Mtb evolution within the host. Overall, this study represents a tour de force in the analysis of pathogen evolution in vivo and addresses a long-standing question in the field regarding the extent and significance of Mtb diversification.

Minor points for improvement

Estimation of population size underlying SNP emergence

1. The population-level sequencing approach is a major strength of the study, and it raises an interesting quantitative question. Is it possible to estimate, even approximately, the effective bacterial population sizes within individual lesions or tissues from which the observed SNPs arise? Such estimates-whether derived from variant frequencies, mutation rates, or modeling approaches-would provide valuable context for interpreting the likelihood of mutation emergence, the balance between selection and drift, and the observed differences across experimental groups.

The reviewer brings up an interesting question about when intrahost variants emerge in a tissue (i.e., the population size present in a lesion at time of emergence), as population level understanding of the forces that enable (or disallow) intrahost Mtb populations to diversify will have great relevance in defining potentially adaptive traits.

Unfortunately, the sequencing depth (~100-fold) employed in this study and the requirement of a 10% prevalence to be considered as a variant effectively limits our detection limit to the first 3 generations after lesion founding (effectively, 1 bacteria gaining a mutation in a lesion of 9 wild type bacteria), assuming that mutations are neutral and that tissues are not introducing genetic drift through Mtb killing dynamics—which may not necessarily be the case biologically.

Mutations that occur past the first 3 generations would will likely lie at too low of a frequency to have been captured for sequencing and identified in our study. As such, we are reticent to make conclusions on lesion population size without a more representative distribution of early and later arising mutations. However, we envision future work may employ much deeper sequencing of populations to define both early and late mutational events, which we can then leverage to estimate the populations sizes associated with intrahost mutations.

2. Implications for TB vaccination studies. Given the power of this approach to resolve intrahost evolutionary dynamics, it would be valuable for the authors to comment on how similar population-level sequencing strategies could be applied in future studies of TB vaccination. For example, what expectations might one have regarding mutational trajectories, bottlenecks, or selective pressures in vaccinated versus unvaccinated hosts? A brief discussion of how this framework could inform evaluation of vaccine-induced immune pressures would broaden the impact and translational relevance of the work.

We thank the reviewer for their questions and agree that an integrated barcoding and population whole genome sequencing methodology could be applied to understand vaccine-mediated changes in immune response, which is an area of intense focus for the field. One attractive vaccine approach that has been tested in non-human primates and where we have previously applied barcode sequencing is in intravenous BCG vaccination. There, we found that IV-BCG vaccination induces an ability of the host to restrict the number of Mtb bacteria capable of establishing infection, which surprisingly did not require T cells as much as we might have imagined (Simonson et al, J Exp Med, 2025).

To the reviewer's questions, we have the Mtb bacterial gDNA populations from IV-BCG vaccinated animals with breakthrough infections and would like to explore whether there are differences in mutational rate and/or mutation types in vaccination as part of future work. Direct evidence of damage to Mtb's genome could be a biomarker of a subset of tissues that are directly impacting Mtb after vaccination and may offer us an avenue to

discover novel correlates of protection in the face of lesion-to-lesion heterogeneity. We have now edited the discussion to reflect some of these thoughts (lines 313-321).

Reviewer #2 (Comments for the Author):

Overall Assessment:
This study employs a non-human primate (NHP) model with SIV co-infection and ART treatment to conduct large-scale whole-genome sequencing of Mycobacterium tuberculosis (Mtb) isolated from approximately 480 infected tissues. A total of 116 intra-host mutations were identified, and the mutation rates, mutational profiles, and enrichment of mutations in lipid metabolism-related genes under different immune conditions were systematically analyzed. The key strengths of this work include the comprehensive characterization of Mtb intra-host mutational landscapes utilizing multi-tissue sampling in NHP models, and the innovative application of intra-host mutations as secondary barcodes to trace bacterial dissemination in vivo. The findings provide valuable insights for understanding Mtb adaptive evolution under HIV-TB co-infection. The manuscript is well organized and structurally sound; however, several critical aspects require further discussion, clarification, and revision.

Major comments:
1.The limitations of relative mutation rate calculation need to be thoroughly addressed. The authors adopted a fixed generation time of 18 hours for mutation rate estimation and acknowledged that the actual in vivo generation time remains unknown. The elevated mutation rate in the SIV group (Fig. 2A) is attributed to enhanced mutation induced by immune suppression, which may be misleading. This difference could alternatively arise from accelerated bacterial replication and shorter generation time in the SIV group. The marginal trend in CEQ values ($p=0.09$, Fig. 2B) and elevated CFU levels (Fig. 2C) only reflect increased bacterial load, rather than accelerated replication rates. Higher CFU may result from impaired host clearance, as noted by the authors.

We appreciate the reviewer's comments around the uncertainty regarding Mtb's in vivo replication rate in the current manuscript. As noted, differences in these rates will affect calculated mutational rates across treatment groups, so we have addressed this concern in two different ways.

First, we improved the presentation of our original data in a clearer way to explain our intent in Figure 2A to describe the relative mutational frequencies across treatment groups per infection day. However, we appreciate that the use of what looked like absolute rates on the y-axis could be mistaken for an assertion of ground truth mutation rate (often calculated on a per generation basis). To avoid this misconception, we have updated Figure 2A to represent our original data as a fold change in **mutation frequency** normalized by infection day, which involves scaling our original relative mutation rates to the mean value of the Mtb only group (see figure below). This replotting has not changed the data structure or statistical significances, and since mutation frequency per day is

insensitive to exact generation time being used, we feel the original observation that SIV hosts support greater Mtb mutational diversity over infection time remains valid. We have now edited the text (lines 141-145) to better describe the goals of Figure 2A.

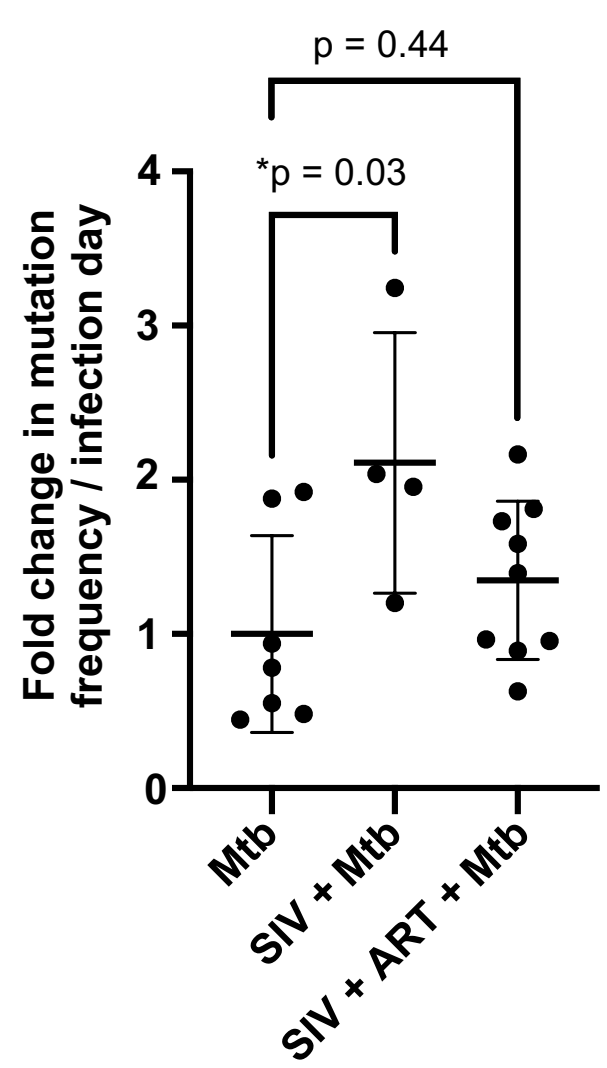

Figure. New Figure 2A replotting the calculated Mtb mutation frequency per infection day as a fold change relative to the Mtb only group.

Second, the referee raises questions around a) the accuracy of using a single fixed replication time to infer Mtb mutation rates across groups, and therefore b) whether the increased number of mutations in SIV-infected hosts can be explained by increased Mtb replication in general (versus increased mutation rate per generation and/or retention of deleterious mutations). We address these concerns with new analyses (see below) that aim to more accurately estimate the in vivo Mtb replication and mutation rate on a per generation basis.

It is recommended that the authors explicitly discuss the potential bias introduced by the fixed generation time assumption, perform sensitivity analyses to validate the robustness of conclusions under different generation time scenarios, and adopt more cautious interpretations regarding the correlation between immune suppression and elevated mutation rates.

We agree that the assumption of a uniform Mtb replication rate across treatment conditions is unlikely; and we may even go further to expect that replication rates will even vary on a NHP basis within groups, given this is a genetically outbred animal model that is known to result in heterogeneous lesion-by-lesion outcomes (Capuano et al., Infect Immun, 2003). Thus, while the referee suggested modeling a range of fixed generation times across groups, we felt the most stringent way to address their concerns and manage animal-to-animal heterogeneity was to use empirical data and estimate NHP-specific Mtb in vivo replication rates. We can then use these replication rates to estimate NHP-specific Mtb mutations rates per generation.

To perform these analyses, we considered that the level of overall Mtb population expansion in a host (i.e., number of population generations) could be estimated if we knew the founding Mtb population size that establishes the infection and the final Mtb burden in the animal (both alive and dead). Fortunately, Diedrich et al., JCI Insight, 2026 collected such data and used this data to approximate our desired values, as follows:

- For the starting Mtb population size, we have previously quantified the number of unique barcoded Mtb strains found in each NHP (figure below), which we have shown in mice and NHPs can be used as a measure of how many Mtb bacteria establish an infection (Simonson et al., Mucos Immun 2025). We now add a plot of the unique barcodes per animal in this study as Supplementary Figure 1B.

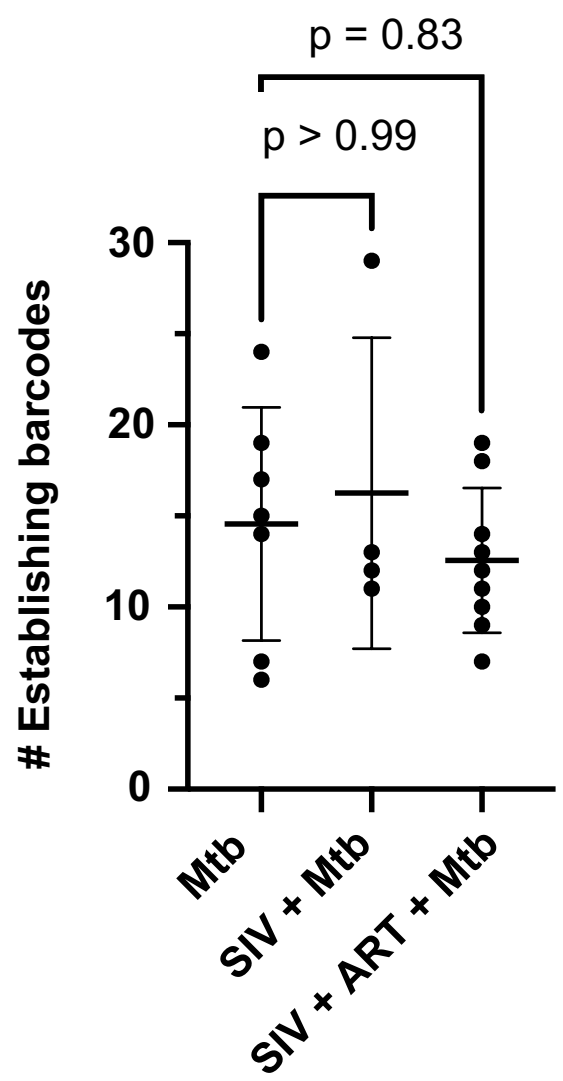

Figure. Number of unique barcoded Mtb strains found in each NHP. Statistical test is a Kruskal-Wallis with Dunn's correction.

- For the final Mtb population size, we used the total number of Mtb chromosomal equivalents (CEQ) summed across all infected tissues (from existing Figure 2B), which approximates the cumulative Mtb population live and dead over the course of infection.

From these data, we then calculate the number of population doublings required to reach the final CEQ value starting with the barcode founder population, using the equation: **X = log2(CEQ / Barcodes)**, where X is the number of population doublings in vivo. These data are presented in new Supplementary Figure 1C (see below).

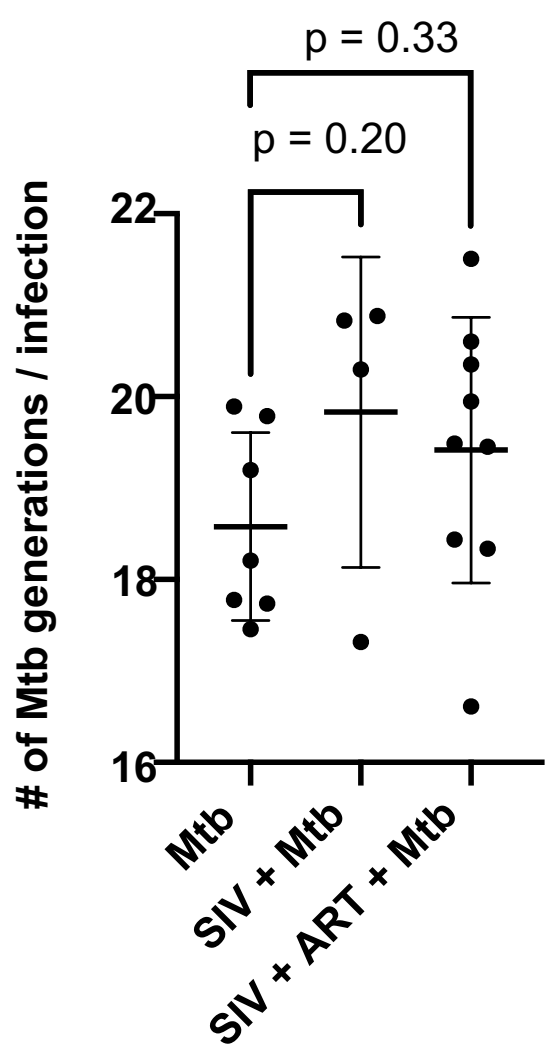

Figure. The calculated number of Mtb generations from founding population size to total CEQ for each NHP. Statistical test is a Kruskal-Wallis with Dunn's correction.

- We can next calculate the mutation rate generation time (g) for each NHP, with the equation: **$g = t / X$** , where the total infection time is divided by number of generations above. These generation times are in new Figure 2D, which finds that there is significantly shorter generation time in the SIV hosts compared to the TB only group. The range of generation times suggests an average population doubling time of 4-5 days over the first 12 weeks of infection.

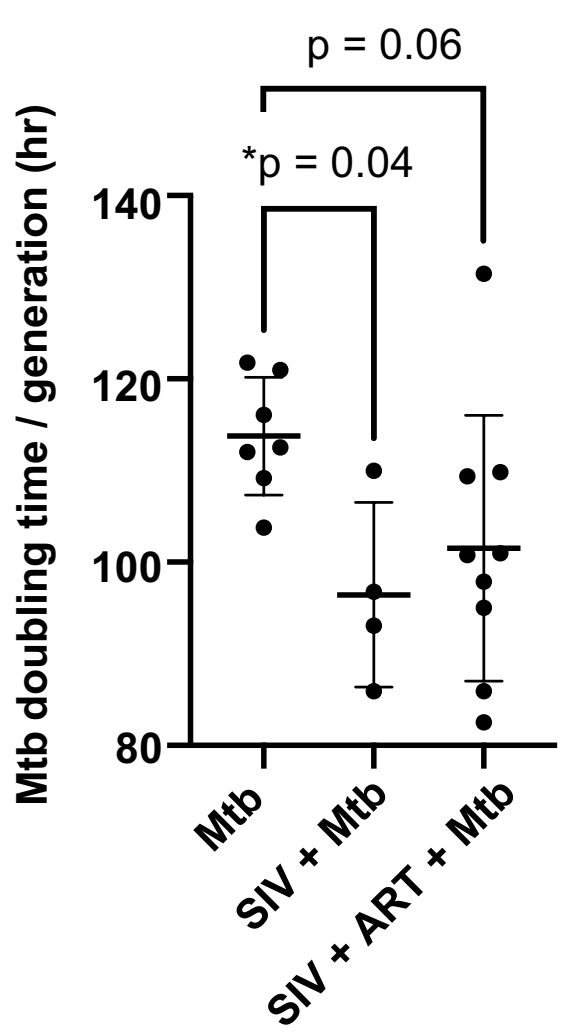

Figure. Calculated overall generation time per NHP. Statistical tests are Kruskal-Wallis tests with Dunn's correction.

- Finally, using these new values, we can calculate the overall in vivo Mtb mutation rate per generation for each NHP (figure below). We find that the TB only group has an average mutation rate of ~2.5×10^{-9} /bp/generation, which is ~10-fold higher than the in vitro mutation rate calculated in Ford et al. Nat Gen 2011 using fluctuation testing of spontaneous rifampicin mutations.

- When we now compare mutation rates across treatment conditions, we do not see a statistical difference in SIV-coinfected hosts compared to Mtb only animals. We interpret these new results—as the referee suggested—that increased Mtb overall replication (presumably due to immunosuppression) likely explains the increased mutational frequencies observed in SIV-infected NHPs.

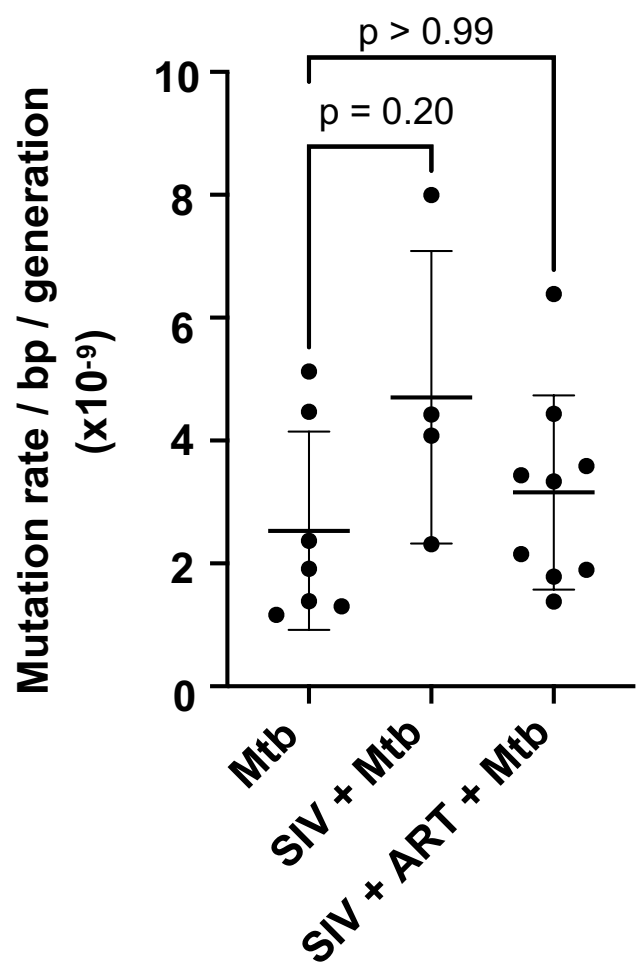

Figure. Re-calculated Mtb mutation rates per Mtb generation. Statistical tests are Kruskal-Wallis tests with Dunn's correction.

As a result of the new analyses above, we have updated the manuscript to include updated Figure 2A, and new Figure 2D and Supplementary Figures 1B-D. We have also revised our conclusions in the results and discussion to highlight that increased replication appears to explain higher SIV mutational frequencies (lines 151-165, 196-297). We have also added the new equations and calculations to the methods (lines 502-513).

2.The causal relationship between oxidative stress-associated mutations and host immune pressure is insufficiently validated. The authors attribute C>T/G>A transitions to oxidative damage induced by host immune responses, supported by reduced bacterial load in mutation-origin tissues (Fig. 2D). However, several concerns remain. First, although C>A artifacts were excluded, potential oxidative damage introduced during tissue processing and DNA extraction has not been assessed, and appropriate technical replicates or control measures are lacking.

The reviewer raises an important point around the causality with which we can conclude that C>T/G>A mutations can be linked to immune pressure (versus artefacts that appear during tissue preparation or genomic DNA extraction).

First, they ask about technical replicates to assess the impact of tissue homogenization or gDNA extraction protocols in introducing mutations. Unfortunately, the sampling strategy in the original study performed tissue homogenization and gDNA extraction on the entirety of each sample, so we do not have technical replicates with which to perform new

extractions and sequencing comparisons. We have updated the Methods text to clarify the reasons for lack of technical replicates (lines 415- 420).

Second, while we agree that tissue and library preparation can be a driver of gDNA damage (as evinced by seeing some tissues that have very high number of low-frequency mutations that we attribute to technical artefacts), given the random nature of DNA damage, we would expect any mutations arising from DNA damage during tissue processing to occur at low allele frequency in the tissue population. Our current workflow applies a relatively conservative threshold for mutation calling or redact sites for which we have low confidence, as to avoid samples that may be inherently more damaged. Specifically, our first QC step is to remove samples that harbor more mutations (at any frequency) than observed across the vast majority of samples) and to only consider mutations as real if they are above a 10% prevalence threshold (effectively requiring at least 5 different DNA fragments to contain the same mutation to be called a candidate given our minimum 50x sequence depth threshold). Still, we do take the point that there may be false positives in the data, so have updated the methods with this thought (lines 420).

Second, the increased proportion of oxidative mutations in the SIV+ART+TB group is attributed to dysregulated inflammation, without empirical evidence from immunological indicators, such as cytokine profiles, reactive oxygen species measurements, or macrophage activation markers.

As we interpret the concern, the reviewer has two concerns: 1) whether we can identify immunological features in macrophages beyond CFU control to strengthen the association between tissues containing oxidative mutations with host immune attack in general; and 2) demonstrate immune dysregulation in the ART animals with elevated numbers of oxidative mutations.

To address point 1, we conducted a new correlative analysis using published flow cytometry data (Diedrich et al., JCI Insight 2026) from 18 profiled granulomas that we define as origin sites containing Mtb mutations (11 sites containing oxidative mutations (all were C>T/G>A) and 7 sites that contained non-oxidative mutations (not C>T/G>A, C>A/G>T)). Diedrich et al. measured the percentage of myeloid cells (CD11b-positive) in these granulomas and the percentage of myeloid cells expressing various functional markers and cytokines, so we compared these immune features between lesions containing oxidative or non-oxidative mutations.

In new Figure 2E (figure below), we find that there is a significantly lower percent of myeloid cells expressing IL-10 in lesions associated with oxidative mutations vs. non-oxidative lesions. As IL-10 is known to be an immunoregulatory cytokine that can inhibit macrophage activation and Mtb control, this data suggests that that oxidative mutation harboring lesions may have myeloid cells with increased capacity respond to and control Mtb infection, which would track with our existing CFU data in Figure 2E. We have updated

our text to include these data (lines 175-181). We also add this analysis to the Methods (lines 487-489).

Finally, even with this data, we also agree it is difficult to causally establish the link between oxidative mutations and immune pressure without follow-up targeted interventions, which is beyond the current study. We added this caveat to the text in lines 184-187.

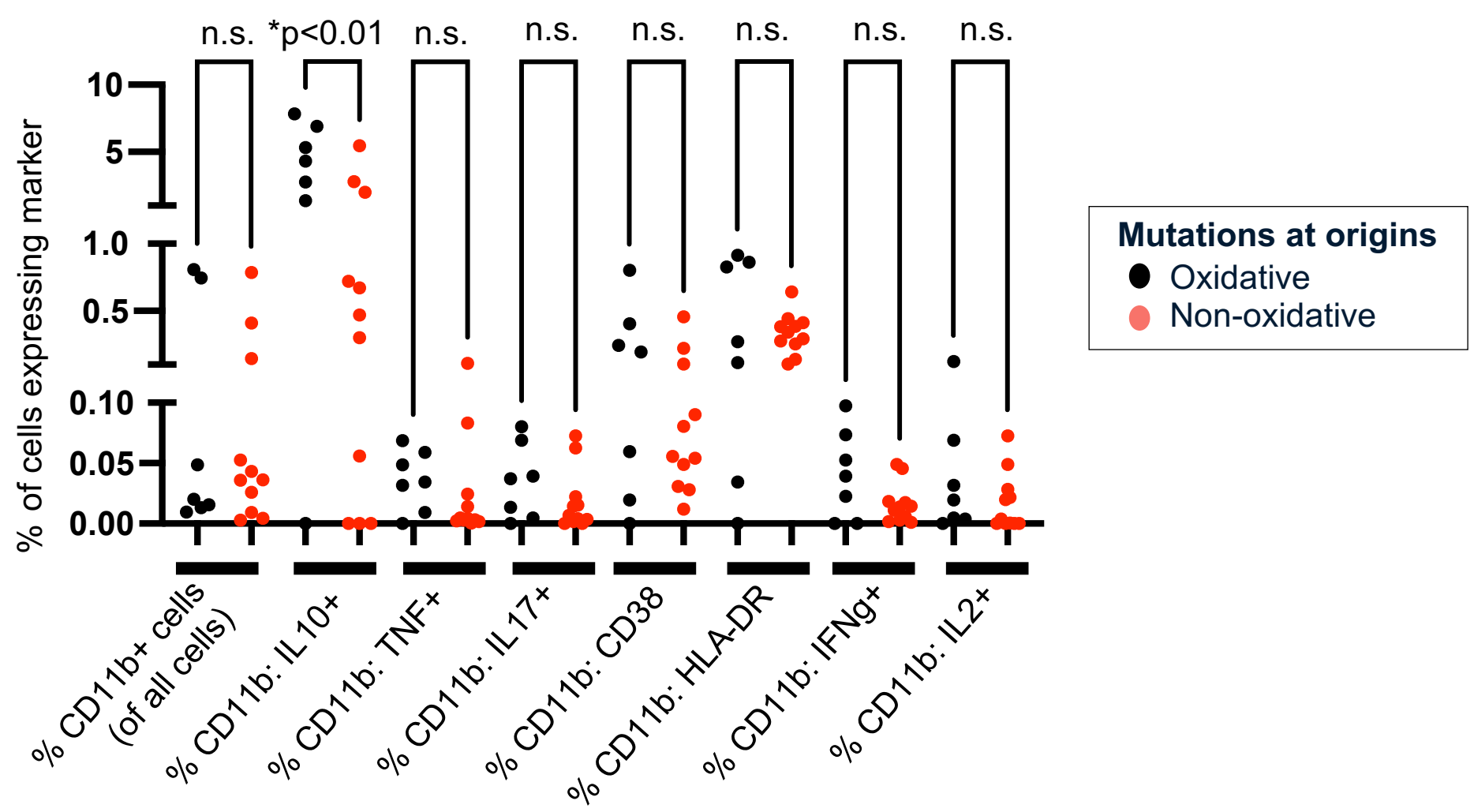

Figure. Flow cytometry data showing the percent of all CD11b-positive myeloid cells that are also expressing cytokines of interest. Statistical testing was performed using a One-way ANOVA with Bonferroni multiple testing correction.

The authors should discuss the potential artifacts from sample processing, moderate their interpretations regarding ART-induced inflammatory stress, and explicitly state that the causal link between oxidative damage and mutational patterns remains hypothetical.

The referee raises a good point about NHP-specific dysregulation across ART treated animals. As our small sample sizes preclude further sub-analysis of the ART treated animals, we have moderated the interpretations regarding dysregulated immune responses in ART animals in lines 194-202. There, we focus on the observed enrichment for oxidative-associated mutations in a subset of ART animals and soften the language around host immune attack as the immediate driver of these patterns.

3.The interpretation of cross-species comparison requires further refinement. The authors compare NHP-derived mutations with a dataset of 55,000 human clinical isolates and highlight that 81% of detected mutations are absent in human strains. It should be noted that human clinical isolates represent globally disseminated lineages filtered by strong transmission bottlenecks. In contrast, most intra-host mutations identified in NHP are

transient and not expected to be captured at the population level. Rather than emphasizing mutation overlap ratios, the discussion should focus on convergent mutations emerging independently in both human and NHP populations, which provide key evidence for adaptive convergence.

We agree with the referee as to the importance of the interhost transmission bottleneck, which may exert a strong purifying selection force on relatively rarer intrahost variants from being shared across individuals. We have accordingly updated the discussion text (line 374-375).

Minor comments:
1.Inconsistent descriptions of sample size exist: "approximately 480 infected tissues" in the abstract and "482 tissues" in the results. Uniform terminology is recommended throughout the manuscript.

We appreciate the reviewer's point and have used the exact number of tissues (482) for statements of sample size in the abstract.

2.The methodological description for Fig. 1C is incomplete. The grey shaded area represents the 95% confidence interval derived from 1,000 simulations; however, it remains unclear whether genomic features such as GC content, repetitive regions, and alignment complexity were adjusted for in the simulation procedures. Supplementary details are required.

We are grateful for the reviewer's comments, which allowed us to discover and fix a plotting error in our original graph in Figure 1C (where we realized that the grey area—which was meant to represent 2 standard deviations from the mean—was actually plotting a single standard deviation). We have now fixed the gray shading in Figure 1C to indicate 2 standard deviations.

Additionally, we have also updated our simulations (as referee requested) to better account for repetitive genomic regions that were masked in our mutation detection workflow but had not been similarly masked during our sampling simulation. Specifically:

- We formally excluded genomic positions that were masked for low 'mappability' in our previous publication (Marin et al., MRA, 2025) and can be found in the following file: https://github.com/maxgmarin/erdman-asm-explore/blob/main/Results/6_PileupMappability_and_HomologyMapping/Erdman_PupMapperOutput_RunGenmapAndPmap_K50E4/ErdmanPolished.circ.Renamed.LowPileupMap.Below1.K50_E4.bed
- We then re-performed the simulation after masking the repetitive sites for a more accurate comparison.

After our new simulation and plotting a 2 standard deviation threshold, we find that one of our genomic windows (1.1-1.2Mb) no longer has more mutations than would be expected

by chance alone. Mutations in the genomic locus spanning 1.8-1.9Mb (where *pks7*, *pks8* and *pks11*genes are located) are still observed more than would be expected. We have now updated text (line 133) and Figure 1C. We also indicate the use of a mask to remove repetitive regions and that no other genomic features were considered in the simulation (see legend for Figure 1C in lines 695-697).

3.The threshold for defining mutation origin tissues is not justified. Mutant allelic frequency below 75% is used to identify origin sites. The authors should provide literature support or explicit rationale for this threshold.

We thank the reviewer for raising this important point. The purpose for the 75% threshold was to identify tissues retaining evidence of both parental and mutant populations. As there is no prior study that has used a combined barcode and unfixed-mutation framework to infer mutation-origin tissues to our knowledge, we elected to use 75% as an operational threshold rather than as a biological boundary, in order to reduce the likelihood of classifying mutant-dominated tissues as origin sites.

We acknowledge that this cutoff is partly subjective; however, it was selected by analogy to the WHO mutation catalogue framework, in which alleles above the 75% frequency are treated as dominant high-confidence mutations, whereas lower-frequency alleles are consistent with mixed populations (Catalogue of mutations in Mycobacterium tuberculosis complex and their association with drug resistance, 2nd ed, 2023). As noted on page 6: "Variants were graded if they occurred at an allele frequency of at least 75% in at least one isolate and if the pDST result was valid."

Further, in accordance with the reviewer's comments, we re-analyzed our variant list to assess how using a more stringent cutoff for fixed mutations would change our origin site designations. Using the data from Supplementary Table 2, we find that defining fixed mutations as those that are >90% allele frequency (i.e., allowing unfixed mutations to be considered if they are 10-90% of reads) yielded the same number of origin sites as using our original 75% cutoff. Effectively, the tissues colonized by a single bacterial strain—but that were not originally considered origins had mutant allele frequencies >95%—which we feel are very likely fixed mutations and may have been carried in from other sites. Thus, while we agree that defining a threshold for origin sites would benefit from future benchmarking studies, we show that using a more stringent definition of fixed mutations would not change our results in this study. We have added the WHO reference and our assessment of a 90% threshold our methods for transparency (lines 474-476).

Reviewer #3 (Comments for the Author):

Chao and colleagues present a fascinating study identifying the intrahost mutational landscape of Mtb infected non-human primates in the context of Mtb-SIV coinfection and

with antiretroviral therapy. The study encompasses a large sequencing study of hundreds of barcoded Mtb infected tissues across 20 NHPs under infection, confection or treatment conditions. From the total mutations identified that emerge during infection, the authors identify subsets of tissue specific mutations versus universal across tissues and across treatment groups. They show a higher mutation rate in SIV infected NHPs, concordant with increased bacterial outgrowth. Gene groups of interest include lipid metabolism and pks genes. Interestingly, the authors also identify NHP-derived mutations, that are independent from clinical isolated from human populations. Overall, this is a thorough and well conducted study that is very timely for the field in identifying early infection mutational events. Importantly, this works also sheds light on dissemination events and various immune pressure encountered during co-infection and treatment.

No major comments.

Minor comments:
-What was the proportion of male and female NHPs in the infection studies, including the new samples? Does sex have any effect on mutational rates across the samples? Curious if this is linked with immune pressures or worth commenting on in the discussion.

The reviewer brings up an interesting point, as sex dimorphism in TB disease outcome is a well-known feature of human infection. In this study, we only had 2 female animals (1 in the TB only group and 1 in the ART arm), which is largely reflective of the sex distribution in the main study (only 4 female animals in the entire study): https://insight.jci.org/articles/view/199314/sd/1 .

These numbers preclude any formal statistical analysis, so have we elected not to include these analyses in the revised manuscript. However, for referee reference, we have highlighted (in magenta) where the female NHPs fall on several of our outcomes metrics. In the Figures below, we observe that female animals have outcomes (e.g. mutation rate, CEQ and frequency of C>T/G>A mutations) that intermingle with the distribution from male animals, which does not suggest a strong sex bias, at least by these metrics.

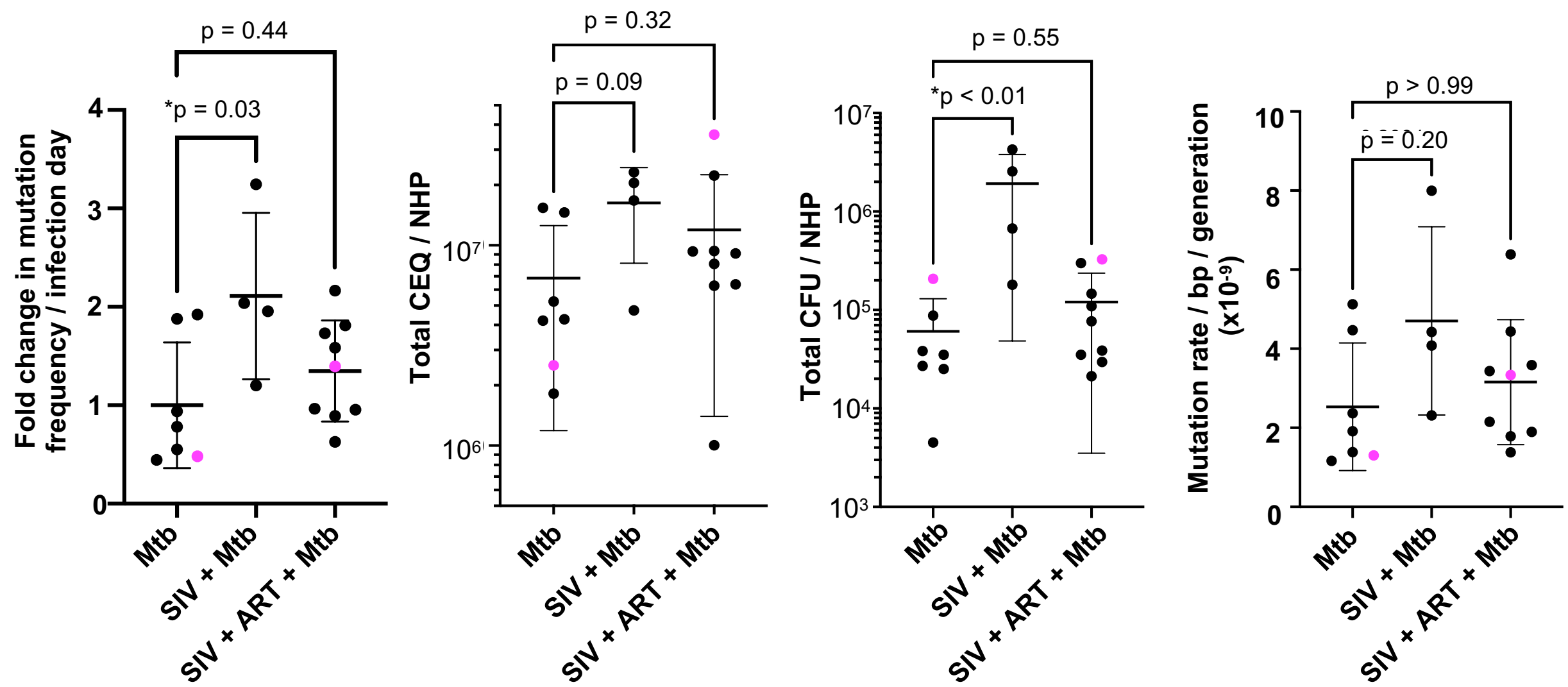

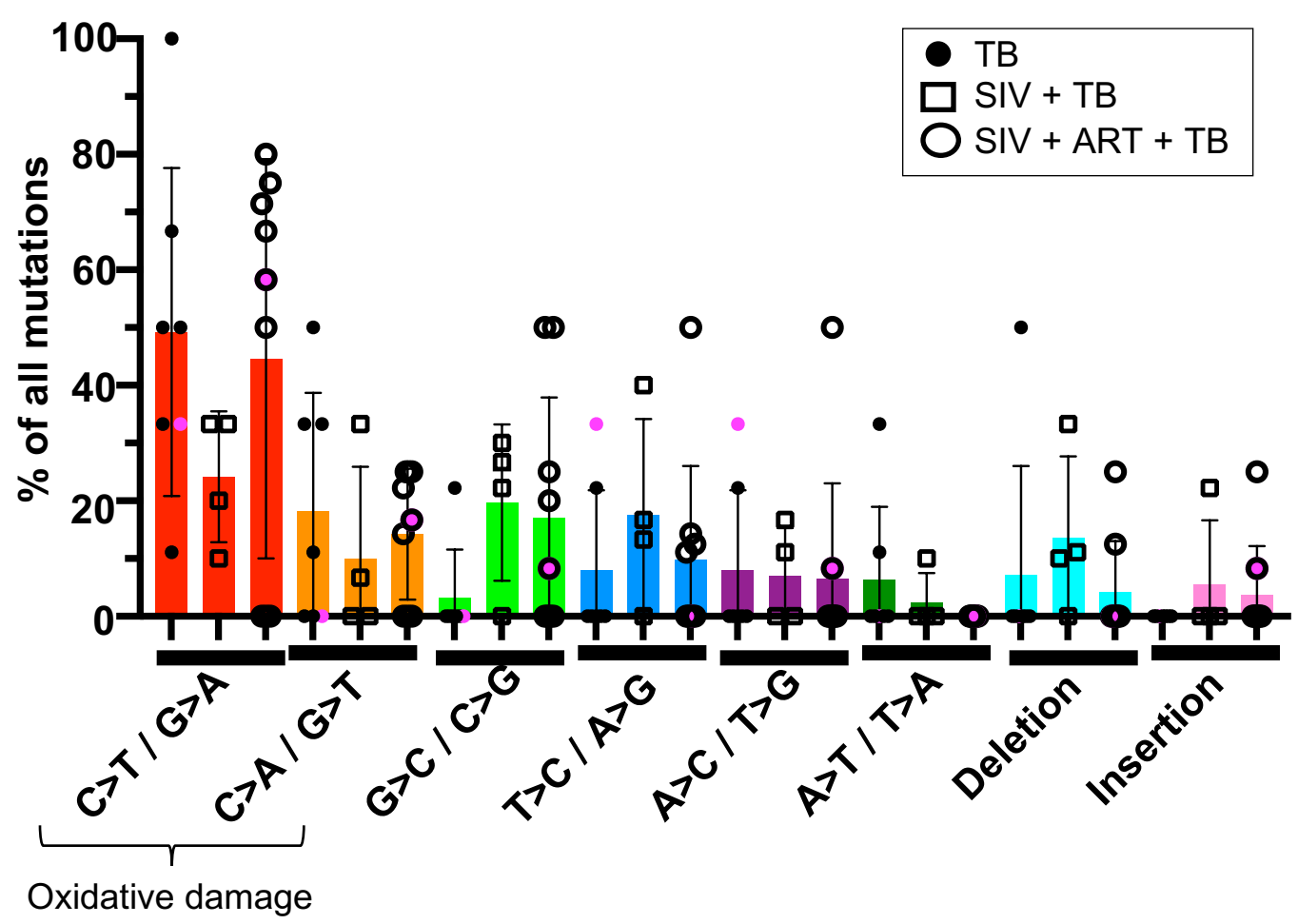

-Even though these are curated from a previous study, recommend the authors include the IACUC statement in methods (as this study includes several new samples).

We appreciate the suggestion from the reviewer and have now added an IACUC approval statement for the original NHP study to the methods (lines 390-394).

Re: mSystems00329-26R1 (Population-level genome sequencing reveals distinct Mycobacterium tuberculosis intrahost mutational trajectories in simian immunodeficiency virus co-infected and antiretroviral treated non-human primates)

Dear Dr. Michael C Chao:

All three reviewers agree on an acceptance.

Your manuscript has been accepted, and I am forwarding it to the ASM production staff for publication. Your paper will first be checked to make sure all elements meet the technical requirements. ASM staff will contact you if anything needs to be revised before copyediting and production can begin. Otherwise, you will be notified when your proofs are ready to be viewed.

Data Availability: ASM policy requires that data be available to the public upon online posting of the article, so please verify all links to sequence records, if present, and make sure that each number retrieves the full record of the data. If a new accession number is not linked or a link is broken, provide production staff with the correct URL for the record. If the accession numbers for new data are not publicly accessible before the expected online posting of the article, publication may be delayed; please contact ASM production staff immediately with the expected release date.

Publication Fees: For information on publication fees and which article types have charges, please visit our website. We have partnered with Copyright Clearance Center (CCC) to collect author charges. If fees apply to your paper, you will receive a message from no-reply@copyright.com with further instructions. For questions related to paying charges through RightsLink, please contact CCC at ASM_Support@copyright.com or toll free at +1-877-622-5543. CCC makes every attempt to respond to all emails within 24 hours.

ASM Membership: Corresponding authors may join or renew ASM membership to obtain discounts on publication fees. Need to upgrade your membership level? Please contact Customer Service at Service@asmusa.org.

Embargo Policy: A press release may be issued as soon as the manuscript is posted on the mSystems Latest Articles webpage. The corresponding author will receive an email with the subject line "ASM Journals Author Services Notification" when the article is available online.

Cover Image Submissions: If you would like to submit a potential Cover Image, please email a file and a short legend to msystems@asmusa.org. Please note that we can only consider images that (i) the authors created or own and (ii) have not been previously published. By submitting, you agree that the image can be used under the same terms as the published article. Image File requirements: TIF/EPS, 7.5 inches wide by 8.25 inches tall (at least 2,250 pixels wide by 2,475 pixels tall), minimum 300 dpi resolution (600 dpi preferred), RGB, and no figure elements, e.g., arrows or panel labels. The legend should be a short description of the image, 1-2 sentences recommended. Please download and use this interactive template in Adobe to ensure that your proposed cover image meets our size requirements (https://journals.asm.org/pb-assets/pdf-text-excel-files/ASM-Interactive-Sizing-Cover-Template-1715689791.pdf).

Thank you for submitting your paper to mSystems.

Sincerely,
Min Yue
Editor
mSystems

Reviewer #2 (Comments for the Author):

The authors addressed all the concerns properly.

Reviewer #3 (Comments for the Author):

The authors have addressed all reviewer comments and revised the manuscript. No further comments
